# Cell cycle-dependent organization of a bacterial centromere through multi-layered regulation of the ParABS system

**DOI:** 10.1371/journal.pgen.1010951

**Published:** 2023-09-21

**Authors:** Jovana Kaljević, Coralie Tesseur, Tung B. K. Le, Géraldine Laloux

**Affiliations:** 1 de Duve Institute, UCLouvain, Brussels, Belgium; 2 John Innes Centre, Department of Molecular Microbiology, Norwich, United Kingdom; Max Planck Institute for Terrestrial Microbiology: Max-Planck-Institut fur terrestrische Mikrobiologie, GERMANY

## Abstract

The accurate distribution of genetic material is crucial for all organisms. In most bacteria, chromosome segregation is achieved by the ParABS system, in which the ParB-bound *parS* sequence is actively partitioned by ParA. While this system is highly conserved, its adaptation in organisms with unique lifestyles and its regulation between developmental stages remain largely unexplored. *Bdellovibrio bacteriovorus* is a predatory bacterium proliferating through polyploid replication and non-binary division inside other bacteria. Our study reveals the subcellular dynamics and multi-layered regulation of the ParABS system, coupled to the cell cycle of *B*. *bacteriovorus*. We found that ParA:ParB ratios fluctuate between predation stages, their balance being critical for cell cycle progression. Moreover, the *parS* chromosomal context in non-replicative cells, combined with ParB depletion at cell division, critically contribute to the unique cell cycle-dependent organization of the centromere in this bacterium, highlighting new levels of complexity in chromosome segregation and cell cycle control.

## Author summary

The precise distribution of genetic material to the progeny is essential for all living organisms, and the ParABS system is critical for this process in most bacteria. Our study provides novel insights into the regulation of this system in a predatory bacterium that exhibits a non-canonical cell cycle, in which the chromosome is copied and segregated multiple times during growth inside a prey bacterium. Our work reveals the subcellular dynamics and multi-level regulation of the ParABS system in this bacterium, which results in the unique cell-cycle dependent assembly of the segregation complex at the chromosomal centromere-like region. Our findings provide a deeper understanding of the adaptation of the ParABS system across bacterial species and developmental stages.

## Introduction

The precise segregation of genetic material during cellular division is fundamental across all domains of life. In eukaryotes, sister chromosomes are tethered to the spindle during mitosis via kinetochores, which are protein complexes that assemble at the chromosomal centromeres. The kinetochores serve as attachment points for the spindle to pull chromosomes apart. Bacteria exploit a conceptually analogous strategy to partition copies of their chromosomal DNA into future daughter cells [1].

In most bacterial lineages, chromosome segregation is achieved by the active partitioning of the duplicated chromosomal origins (*oriC*) via the ParABS system [2,3]. The leading player in this system, the DNA-binding CTPase ParB, loads onto the chromosome by binding to *parS* sites - short palindromic sequences, usually found near the *oriC* [2,4–8]. Binding of ParB-CTP on *parS* allows ParB to spread away and cover adjacent DNA, leading to the accumulation of ParB on a bacterial centromere-like region and the formation of a higher-order nucleoprotein structure known as the partitioning complex [9–14], reminiscent of the eukaryotic kinetochore. CTP hydrolysis favors the opening of the ParB clamp and its removal from the DNA, replenishing the pool available for binding on one or more *parS* sites [11,14,15]. Upon initiation of chromosome replication at *oriC*, a second partitioning complex assembles on the duplicated centromere, which will be segregated by the ParABS system. Briefly, ParB·*parS* interacts with ParA, a protein that dimerizes in its ATP-bound form and associates non-specifically with the DNA, therefore coating the entire nucleoid [16,17]. The partitioning complex stimulates the ATPase activity of ParA^ATP^, displacing it from the *oriC*-proximal edge of the DNA-bound “cloud” and generating a ParA^ATP^ gradient towards the opposite cell pole. Repeated ParA-ParB interactions drive the movement of the centromere towards the highest ParA^ATP^ concentration and across the cell, followed by the rest of the sister chromosome [3,18–21].

In several organisms, this process is enhanced by specific landmark polar proteins that either anchor the chromosomal *oriC* [22–25] or sequester the released ParA^ADP^ monomers to maintain the ParA^ATP^ gradient [26–28]. These mechanisms play a role in ensuring the unidirectionality of chromosome segregation and establishing polarity in future daughter cells [29]. Bacteria also evolved diverse strategies to precisely couple the positioning and timing of cell constriction with the segregation of either *oriC* [30] or the chromosomal terminus (*ter*) [31,32]. Consequently, segregation of bacterial chromosomes is tightly coordinated in time and space with cell cycle progression [33]. While ParABS was demonstrated to be essential for survival in a few bacterial species [34–37], its inactivation or depletion usually leads to pleiotropic phenotypes emanating from impaired *oriC* segregation, including aberrant chromosome numbers but also cell cycle progression and cell division defects [3].

ParABS systems translocate chromosomes in a variety of organisms with vastly different lifestyles, cell cycles, and differentiation programs. Despite significant advances in understanding the subcellular dynamics, biochemistry, and interactions of the ParABS components during chromosome segregation [3,38], the adaptation of this highly conserved system across species and between developmental stages remains unclear. Only a few investigations have examined the regulatory mechanisms tuning the ParABS system [39]. For example, studies have highlighted the importance of balancing the ParA and ParB levels to ensure correct cell cycle progression in *Caulobacter crescentus* [34], the ParA (Soj)-dependent transcriptional modulation of the *parAB* operon in *Bacillus subtilis* [40] and the developmental regulation of *parA* and *parB* expression that couples the segregation of the linear chromosome with the mycelial lifestyle of *Streptomyces coelicolor* [41]. Except for the latter, most available data on ParABS-dependent chromosome partitioning come from bacteria that proliferate classically through vegetative growth and binary division (one mother cell generating two daughter cells). However, many species possessing a ParABS system rely on relatively complex cell cycles involving non-binary division events, generating larger and sometimes variable numbers of progeny from a polyploid mother cell (*i*.*e*., *Cyanobacteria*, *Bdellovibrionata*, and *Actinobacteria*) [42–44]. The exploration of how the ParABS system partitions numerous copies of the chromosome during the intricate cell cycle of these organisms is still limited.

**Fig 1:**
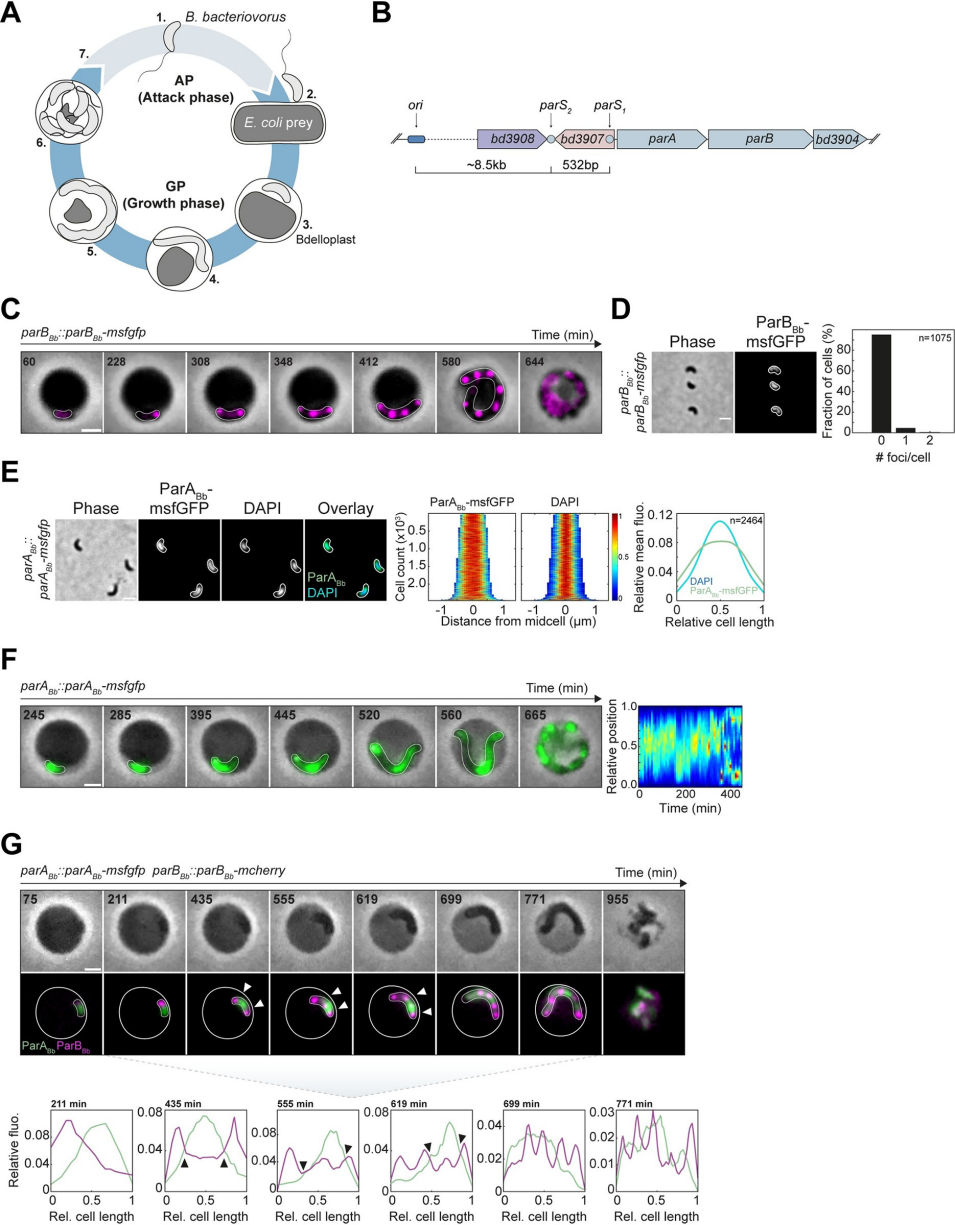
Subcellular localization of the ParABS system during the *B*. *bacteriovorus* cell cycle. (A) Schematics of the *Bdellovibrio bacteriovorus* cell cycle. Numbers indicate key steps in the cycle: 1. Freely-swimming attack phase (AP) cells, 2. Attachment of *B*. *bacteriovorus* to its prey, 3. *B*. *bacteriovorus* resides in the periplasm of the prey, which is now called bdelloplast, 4. Filamentous growth and consumption of prey content, 5. Pre-divisional state, 6. The non-binary division of the mother cell generates an odd or even number of daughter cells, which mature before 7. escaping the prey remnants and resuming the cell cycle. Attack phase (AP) and growth phase (GP) represent the non-replicative and replicative stages of the cell cycle, respectively. (B) Chromosomal context of the *parABS* system in the WT *B*. *bacteriovorus* genome. The *parAB* operon is shown in blue. *bd3908* encodes a putative rRNA methyltransferase*; bd3907* encodes an unknown protein*; parA* and *parB* are *bd3906* and *bd3905*, respectively; *bd3904* encodes a bactofilin homolog. The genomic distance between chromosomal *ori* and both *parS* sites is indicated. (C) Cell-cycle dependent localization of ParB_Bb_. *B*. *bacteriovorus* strain *parB*_*Bb*_::*parB*_*Bb*_*-msfgfp* (GL1654) was mixed with *E*. *coli* prey and imagined in time-lapse after 60 min with 8-min intervals. Overlays of phase contrast and fluorescence images of selected time points are shown; multiple well-separated ParB_Bb_-msfGFP foci appear over time; in this example, ParB_Bb_-msfGFP foci disappear at time point 664 min. The time-lapse is shown in S1 Video. (D) Endogenous ParB_Bb_ does not form an *oriC*-bound focus during AP. Representative phase contrast and fluorescence images of the *parB*_*Bb*_::*parB*_*Bb*_*-msfgfp* strain (GL1654) for representative AP cells; histogram representing the percentage of cells with zero, one, or two ParB_Bb_-msfGFP foci in the same strain. (E) ParA_Bb_ is nucleoid bound. Left to right: representative phase contrast and fluorescence images of AP cells of the *parA*_*Bb*_::*parA*_*Bb*_*-msfgfp* strain (GL2134) stained with DAPI; demographs of the corresponding fluorescent signals in the same cells sorted by length and oriented based on signal intensity; heatmaps represent relative fluorescence intensities; mean pole-to-pole profiles of relative fluorescence intensity of the corresponding signals in the same cells. (F) Endogenous ParA_Bb_ moves dynamically along the growing predator filament. From left to right: *B*. *bacteriovorus* strain *parA*_*Bb*_::*parA*_*Bb*_*-msfgfp* (GL2134) was mixed with prey and imaged in time-lapse after 80 min with 5-min intervals; left: overlay of phase contrast and fluorescence images, time points are shown in min; right: kymograph of the ParA_Bb_-msfGFP signal along the cell length for the same cell until time point 445 min. The time-lapse is shown in S2 Video. (G) The dynamic interplay between ParB_Bb_ and ParA_Bb_. *B*. *bacteriovorus* strain *parA*_*Bb*_::*parA*_*Bb*_*-msfgfp parB*_*Bb*_::*parB*_*Bb*_*-mcherry* (GL2154) was mixed with prey and imagined in time-lapse after 75 min with 8-min intervals. Top: phase contrast; bottom: overlay of ParB_Bb_-mCherry and ParA_Bb_-msfGFP signals. Arrowheads point to a ParB_Bb_ focus on the edge of a ParA_Bb_-msfGFP cloud. Bottom: pole-to-pole profiles of relative fluorescence intensity of the corresponding fusions in the same cells; arrowheads point to ParB_Bb_ foci at the edge of ParA_Bb_-msfGFP clouds. The time-lapse is shown in S3 Video. Scale bars are 1 μm. n indicate the number of cells analyzed in a representative experiment. *B*. *bacteriovorus* and bdelloplasts outlines in panels C, F, G were drawn manually based on the phase contrast images. All experiments were performed at least twice. See also S1 Fig.

The obligate predatory bacterium *Bdellovibrio bacteriovorus* proliferates through filamentation and non-binary division inside the envelope of other diderm bacteria. Its cell cycle comprises two main phases determined by its presence outside or inside a prey **(Fig 1A)** [45,46]. The first phase, called attack phase (AP), corresponds to a G1 cell cycle stage during which *B*. *bacteriovorus* do not replicate their single circular chromosome as they search for prey [47]. Upon contact, predators attach to the prey surface and invade their periplasm, i.e., the space located between the inner and outer membranes [48,49]. Upon a G1-S transition, *B*. *bacteriovorus* starts the second stage of its cell cycle called the growth phase (GP), during which the cell elongates and copies its DNA multiple times. A first round of chromosome replication and segregation, which starts from the *oriC* localized at one cell pole [47], is followed by the asynchronous firing of additional replication rounds using the *oriC* of multiple chromosome copies as a template [47,50]. The newly synthesized chromosomal origins segregate progressively [47], unlike in *Streptomyces*, which also grows as polyploid filaments but uncouples DNA replication during vegetative growth from the ParABS-dependent segregation of multiple chromosome copies at sporulation. The filamentous and polyploid mother *B*. *bacteriovorus* cell eventually divides by synchronous constriction at multiple locations along the cell body, releasing a variable, odd, or even number of offspring [47,50–52]. The extent of the GP and the number of predator daughter cells is determined by the size of the prey [53], which remains a closed nest as it is being digested by *B*. *bacteriovorus*, until newborn predators break the prey open to resume their predatory cycle [54].

In contrast to all other ParB homologs involved in chromosome segregation that always localize at the *parS* site [7,35,37,55–65], ParB in *B*. *bacteriovorus* (ParB_Bb_) does not mark the centromere throughout the cell cycle. Our previous report showed that fluorescently tagged ParB_Bb_, produced at native or constitutive levels, accumulates near *oriC* only after the initiation of chromosome replication, while the protein displays a diffuse localization in the predator progeny [47]. The constitutive production of ParB_Bb_ disturbs the progressive segregation of chromosomal copies, resulting in newborn *B*. *bacteriovorus* with aberrant cell length, *oriC* numbers, and nucleoid size. Thus, although ParB is critical for proper *oriC* partitioning and cell cycle progression in *B*. *bacteriovorus*, the formation of a ParB·*parS* higher order structure at the centromere-like region is prevented during the non-proliferative stage of the predatory cycle [47]. The underlying mechanism behind this cell cycle-dependent on-off behaviour of ParB_Bb_ is unknown. Furthermore, the subcellular dynamics of ParA have not been explored in bacteria that segregate multiple pairs of sister chromosomes during their vegetative cell cycle. An in-depth view of the ParABS system throughout the lifecycle of *B*. *bacteriovorus* is crucial to better understand how it operates in species with non-binary proliferation. With its intricate chromosome dynamics and unique centromere organization during the cell cycle, *B*. *bacteriovorus* represents a compelling model to uncover new levels of regulation in the highly conserved ParABS system.

Here we provide key insights into the ParABS system in *B*. *bacteriovorus*, by monitoring the subcellular localization, expression, and protein levels of ParA_Bb_ and ParB_Bb_ during all stages of the predatory cell cycle. Moreover, we characterize ParB_Bb_ CTPase and *parS* binding activities. Our data reveal multiple regulation layers in the ParABS system of this bacterium. We show that ParA_Bb_ and ParB_Bb_ protein levels fluctuate differently during the cell cycle despite being expressed from the same operon, their fine balance being critical for proper cell cycle progression. Uniquely, the genomic context of the *parS* sites during the non-replicative stage, combined with ParB_Bb_ protein depletion when the mother cell divides, play a critical role in the unique cell cycle-dependent organization of the centromere.

## Results

### The ParABS system is critical for survival and exhibits specific localization patterns in *B*. *bacteriovorus*

The *B*. *bacteriovorus* genome encodes homologs of ParA and ParB and harbors two *parS* sites near the chromosomal origin, indicating the presence of a ParABS system for chromosome segregation **(Fig 1B)**. All our attempts to individually delete *parA*_*Bb*_ and *parB*_*Bb*_ were unsuccessful (86 and 90 screened recombinants, respectively, see Methods), underscoring that the ParABS system is likely essential in this organism under the tested conditions. We have shown previously that ParB_Bb_ labeled with the fluorescent protein mCherry localizes as foci that mark the multiple copies of chromosomal *oriC* during their partitioning in the growth phase (GP) [47], consistent with the conserved role of ParB in chromosome segregation. However, the localization of ParB_Bb_ appeared to be cell-cycle regulated in *B*. *bacteriovorus* [47], whereas all ParB proteins described in other species permanently cluster at the centromere [7,35,37,55–65]. Using a ParB_Bb_-msfGFP fusion produced as a single copy from the native chromosomal locus, we confirmed that ParB_Bb_ forms foci only during the growth phase of the predator cell cycle **(Fig 1C,** S1 **Video**). Conversely, the ParB_Bb_-msfGFP fluorescence signal was weak and diffuse in the cytoplasm in non-replicating cells, i.e., in the free attack-phase (AP) cells (**Fig 1D**) or during the time window corresponding to the G1-S transition upon prey invasion [47] (**Fig 1C)**.

To obtain more insights into the functioning of the ParABS system during the cell cycle of *B*. *bacteriovorus*, we constructed a functional ParA_Bb_-msfGFP fusion **(S1A Fig)** produced natively as a single copy and monitored its subcellular localization. We found that ParA_Bb_-msfGFP co-localized with the nucleoid in non-replicative cells (**Fig 1E**), in agreement with the non-specific DNA binding of ParA proteins [16,17]. Unlike in other species with a polarly localized *oriC*, ParA_Bb_-msfGFP localization was not visibly biased towards a particular cell pole, which we were able to distinguish using the invasive cell pole marker RomR-mCherry [47,66] **(S1B Fig**). During the *B*. *bacteriovorus* proliferative phase inside the prey, ParA_Bb_-msfGFP displayed a dynamic localization pattern reminiscent of the DNA-bound ParA “cloud” characterized in other organisms (**Fig 1F,** S2 **Video**), which pulls apart duplicated ParB·*parS* partitioning complexes via repeated ParA-ParB interactions [19,67]. In addition, we detected an interaction between ParA_Bb_ and ParB_Bb_ in a bacterial two-hybrid assay (**S1C Fig**) and in a POLAR recruitment assay (**S1D–S1F Fig**) [68], suggesting a similar interplay between these proteins in *B*. *bacteriovorus*. However, in contrast to bacteria that segregate only two copies of their chromosome, several ParA_Bb_-msfGFP accumulations were present at the same time and moved dynamically along the filamentous predator cell, shifting from one subcellular region to another (**Fig 1F,** S2 **Video**). Using a strain carrying both fluorescently labeled ParA_Bb_ and ParB_Bb_ (**S1G Fig**), we observed that ParB_Bb_-mCherry foci were mainly located at the edges of ParA_Bb_-msfGFP clouds **(Fig 1G, arrowheads,** S3 **Video**). This complex localization pattern is consistent with the idea that the *B*. *bacteriovorus* ParABS system is adapted to drive the simultaneous or sequential segregation of multiple chromosome copies.

### A fine-tuned balance of parA_Bb_ and parB_Bb_ expression underlies proper chromosome segregation and cell cycle progression in *B*. *bacteriovorus*

**Fig 2:**
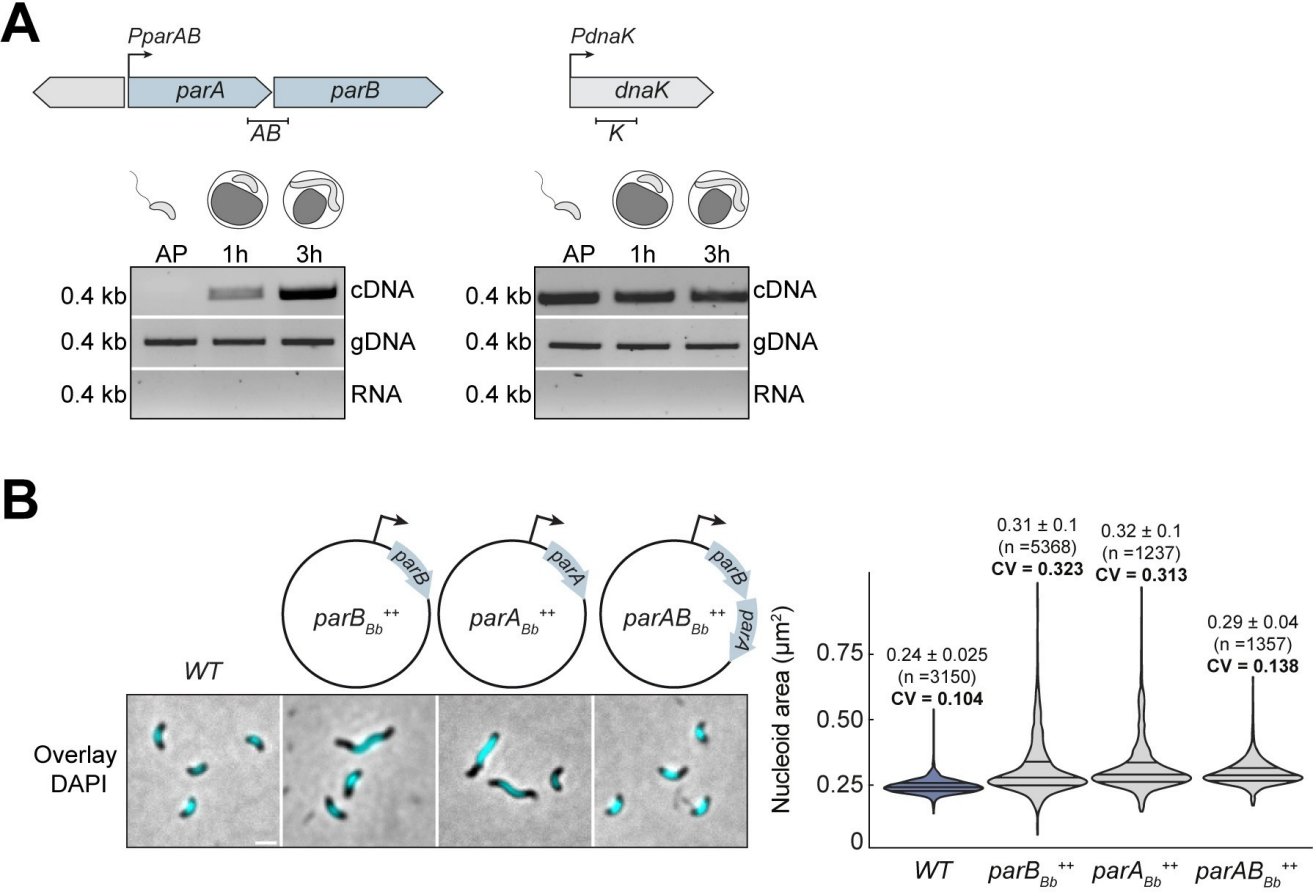
Biphasic expression of the *parAB*_*Bb*_ operon during the *B*. *bacteriovorus* cell cycle is critical for proper chromosome segregation. (A) Biphasic expression of the *parAB*_*Bb*_ operon. Top: schematic representation of the *B*. *bacteriovorus parAB* and *dnaK* (housekeeping gene) genomic context; graphical illustrations depict the cell cycle stages corresponding to the indicated time points. The fragments corresponding to the *parA-parB* junction or inside the *dnaK* gene amplified by RT-PCR are indicated below (*AB*, 400 bp; *K*, 400 bp). RNA samples were isolated from wild-type *B*. *bacteriovorus* in AP, 1 h, and 3 h after mixing with prey. Bottom: agarose gel analysis of the indicated fragments amplified by PCR from cDNA (top panel), genomic DNA (middle panel, positive control), and total RNA (bottom panel, negative control, ensuring RT-PCR bands result from the cDNA and not from potential DNA contamination in the RNA sample). Molecular weight markers (in kb) are indicated on the left. (B) Balanced *parA*_*Bb*_:*parB*_*Bb*_ expression is essential for proper chromosome partitioning. Left: phase contrast and fluorescence images of representative AP cells stained with DAPI. The schematics illustrate the presence of a plasmid allowing the constitutive expression (from the P*nptII* promoter) of the indicated gene(s) *parB*_*Bb*_ (GL1261), *parA*_*Bb*_ (GL1460), or both (GL1004) in an otherwise *WT* background. Cells with abnormal nucleoid areas are observed when *parA*_*Bb*_ (*parA*_*Bb*_^*++*^) or *parB*_*Bb*_ (*parB*_*Bb*_^*++*^) is overexpressed, but to a lower extent when both are overexpressed (*parAB*_*Bb*_^*++*^). Scale bar, 1μm. Right: violin plot of cell length, cell area, and nucleoid area distributions in the same cells. The lines indicate the 25, 50, and 75 percent quantiles from bottom to top. Mean, standard deviation and coefficient of variation (CV) values are shown on top of the corresponding plot. n indicate the number of cells analyzed in a representative experiment. All experiments were performed at least twice. See also **S2 Fig**.

Remarkably, the ParB_Bb_-associated fluorescence seemed to drop at the end of the cell cycle, unlike ParA_Bb_-msfGFP (see the last time points in **Fig 1C**, **1F**). Thus, our data suggest that these key players of the ParABS system undergo an unknown regulation resulting in distinct changes in protein abundance throughout the predatory cell cycle. To delve deeper into the cell-cycle control of the ParABS system in *B*. *bacteriovorus*, we first investigated the regulation of its expression. RT-PCR shows that *parA*_*Bb*_ and *parB*_*Bb*_ are part of the same operon **(Figs 2A and S2A)**. Furthermore, their expression is cell-cycle regulated as the corresponding mRNA was detected mainly in GP and not in AP **(Figs 2A and S2A)**, in agreement with previous reports [69,70].

Consistent with the significance of the cell cycle-dependent *parAB*_*Bb*_ expression in *B*. *bacteriovorus*, we previously found that the constitutive expression of *parB*_*Bb*_ (*parB*_*Bb*_^++^) negatively impacts cell cycle progression and chromosome segregation [47]. Altering the native *parA*_*Bb*_ expression similarly impaired cell cycle progression and *ori* partitioning, mimicking the *parB*_*Bb*_^++^ phenotype (**Figs 2B and S2B–S2C**). Remarkably, simultaneous constitutive expression of both *parA*_*Bb*_ and *parB*_*Bb*_ (from the same promoter) decreased the severity of the single overexpression phenotypes (**Figs 2B and S2B–S2D**). Thus, our data show that the ParABS system is under transcriptional or post-transcriptional control during the *B*. *bacteriovorus* cell cycle, resulting in phase-dependent abundance of the transcript encoding both *parA*_*Bb*_ and *parB*_*Bb*_. This level of regulation is crucial for proper chromosome segregation and cell cycle progression, likely by contributing to a fine balance between both partners.

### The protein levels of ParA_Bb_ and ParB_Bb_ vary differently during the predatory cell cycle

**Fig 3:**
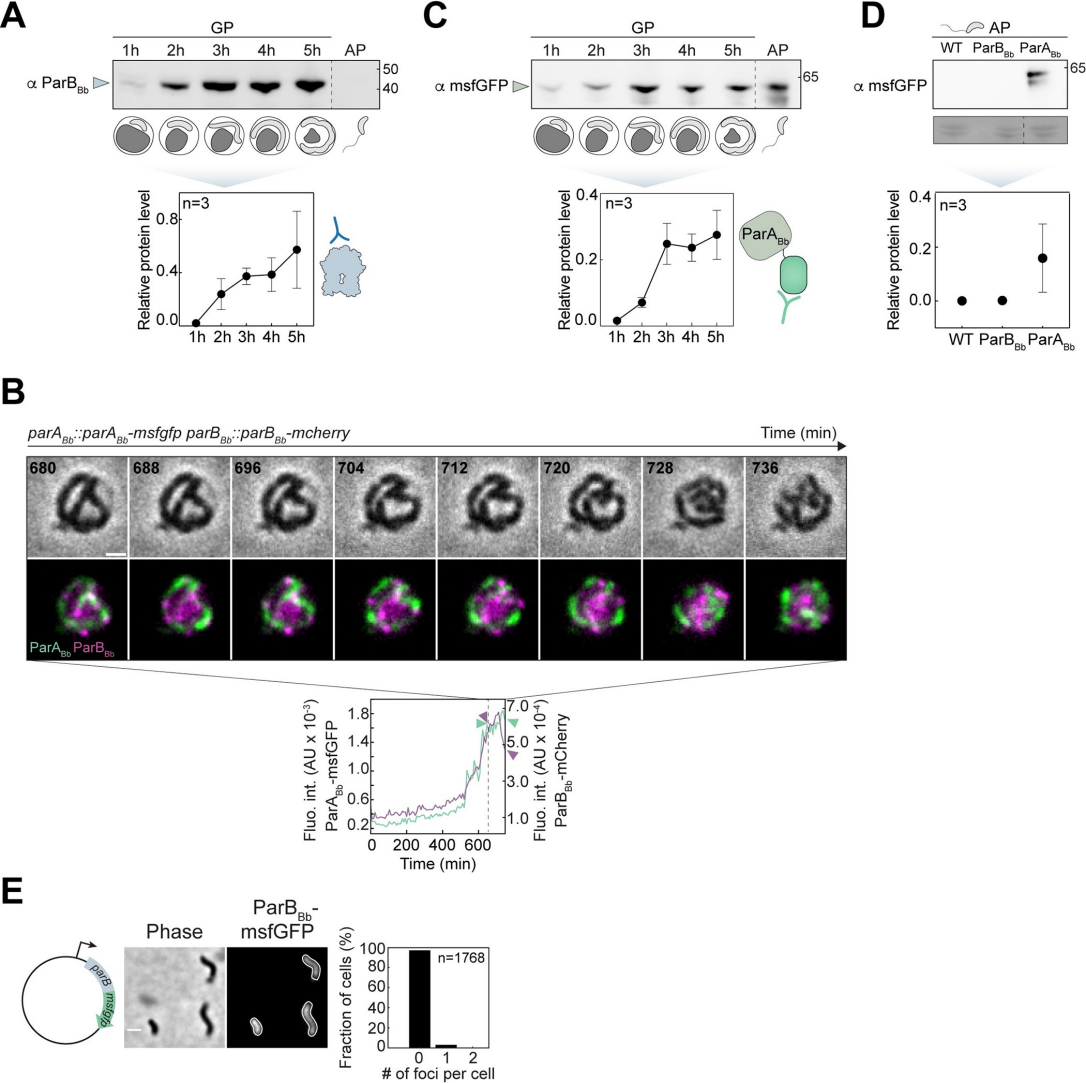
The levels of ParB_Bb_ and ParA_Bb_ vary differently during the cell cycle. (A) ParB_Bb_ protein levels are cell-cycle dependent. Western blots of whole-cell protein extracts from *WT B*. *bacteriovorus* were probed with an anti-ParB_Bb_ antibody, as represented in the schematics. WT protein samples were isolated at time points throughout the predatory cell cycle: 1h, 2h, 3h, 4h, 5h after mixing with prey (GP), and AP. All samples were run on the same gel; for enhanced figure clarity, sample positions have been rearranged, as indicated by the dashed line. Graphical illustration of the cell cycle stages corresponding to the indicated time points. Arrowhead points to ParB_Bb_ (detected only during GP). Ponceau staining served as a loading control and quantification from full lanes (displayed in **S6**A Fig) was used to normalize band intensities. Quantifications are shown as the means of independent triplicates. Error bars represent standard deviations. All replicates are shown in **S6A Fig**. (B) ParB_Bb_-msfGFP fluorescence intensity level drops during cell division while ParA_Bb_-msfGFP fluorescence intensity levels remain high. *B*. *bacteriovorus parA*_*Bb*_::*parA*_*Bb*_*-msfgfp parB*_*Bb*_::*parB*_*Bb*_*-msfgfp* (GL2154) was mixed with prey and imaged in time-lapse after 75 min with 8-min intervals. Top: overlay of phase contrast and fluorescence images for ParA_Bb_-msfGFP and ParB_Bb_-mCherry for selected late time points; Bottom: mean fluorescence intensities of msfGFP (in green) and mCherry (in magenta) plotted over time for the same cell. The time window corresponding to the images displayed on top starts at the dashed line. ParB_Bb_-mCherry fluorescence intensity decreases during this interval, unlike ParA_Bb_-msfGFP. (C) ParA_Bb_ protein levels do not decrease in the attack phase. Western blots of whole-cell protein extracts from *B*. *bacteriovorus parA*_*Bb*_::*parA*_*Bb*_*-msfgfp* (GL2134) probed with an anti-msfGFP antibody, as represented in the schematics. GL2134 protein samples are isolated at time points throughout the predatory cell cycle: 1h, 2h, 3h, 4h, and 5h after mixing with prey (GP), and AP. All samples were run on the same gel; for enhanced figure clarity, sample positions have been rearranged, as indicated by the dashed line. Graphical illustration of the cell cycle stages corresponding to the indicated time points. Arrowhead points to ParA_Bb_-msfGFP (detected also in the AP). Ponceau staining served as a loading control and quantification from full lanes (displayed in **S6**B Fig) was used to normalize band intensities. Quantifications are shown as the means of independent triplicates. Error bars represent standard deviations. All replicates are shown in **S6B Fig**. (D) Top: Western blots of whole-cell protein extracts from AP cells of *B*. *bacteriovorus* WT, *parB*_*Bb*_::*parB*_*Bb*_*-msfgfp* (ParB_Bb,_ GL1654) and *parA*_*Bb*_::*parA*_*Bb*_*-msfgfp* (ParA_Bb_, GL2134) strains were probed with an anti-msfGFP antibody. Only ParA_Bb_-msfGFP protein is detected in AP. Bottom: quantifications are shown as the means of independent triplicates, normalized as in A and C. Error bars represent standard deviations. All replicates and Ponceau staining are shown in **S6C Fig**. (E) Overproduction of ParB_Bb_-msfGFP does not lead to the *parS-*bound focus in AP. Left: representative phase contrast and fluorescence images of AP cells of a WT *B*. *bacteriovorus* strain constitutively producing ParB_Bb_-msfGFP (GL1003). Right: histogram representing the percentage of cells with zero, one, or two ParB_Bb_-msfGFP foci in the same strain; n indicates the number of cells analyzed in a representative experiment. Scale bars are 1 μm. All experiments were performed at least twice. See also **S3 Fig**.

Since ParA_Bb_ and ParB_Bb_ fluorescence profiles hinted at distinct relative protein abundance despite being expressed from the same transcript, we sought to obtain detailed insights into ParA_Bb_ and ParB_Bb_ protein levels during the cell cycle. Western blot using an antibody against ParB_Bb_ shows that the endogenous (untagged) ParB_Bb_ protein levels vary during the cell cycle, being almost unnoticeable in AP but present in higher amounts in GP (**Figs 3A, S3A and S6A**), mirroring the fluorescence signal of the natively produced ParB_Bb_-msfGFP fusion (**Fig 1C**). Accordingly, single-cell analysis of the ParB_Bb_-msfGFP fluorescence intensity profile over time indicates that ParB_Bb_ proteins accumulate during cell growth but rapidly drop when *B*. *bacteriovorus* divides into multiple daughter cells (**Fig 3B, magenta, S3B Fig**). Notably, the levels of natively produced ParB_Bb_-msfGFP or ParB_Bb_-mCherry protein fusions, detected with the same anti-ParB_Bb_ antibody, fully reflected the endogenous untagged ParB_Bb_ profile during a synchronized *B*. *bacteriovorus* cell cycle **(Figs 3A, S3C and S6A)**, confirming that these fusions are reliable reporters of the native ParB_Bb_ protein.

Unlike ParB_Bb_, ParA_Bb_-msfGFP was detected throughout the cell cycle, including in AP (**Figs 3C–3D, S3D and S6B–S6C**), consistent with the corresponding fluorescence signal (**Fig 1F**). ParA_Bb_-msfGFP fluorescence intensity remained high during the last cell cycle stages, including cell division (**Fig 3B, green, S3E Fig**). These results show that, besides cell-cycle regulated expression, the ParABS system in *B*. *bacteriovorus* is subjected to an additional level of control that results in distinct ParA_Bb_ and ParB_Bb_ protein levels at different cell cycle stages. While *in vitro* studies showed that the ParA:ParB balance is important for efficient partitioning [71,72], this is the first evidence of cell-cycle-dependent changes in the relative levels of ParA and ParB proteins.

### The formation of ParB_Bb_·*parS* complexes is inhibited in non-replicative predator cells

In addition to the sudden decrease of ParB_Bb_ protein levels at the end of the GP, the subcellular localization of ParB_Bb_ fluorescent fusions also changed, shifting from clear foci to diffuse localization **(Figs 1C and 3B)**. However, both events could not be easily distinguished due to their occurrence within a relatively short time window and the low fluorescence of the natively produced ParB_Bb_ fusion at that stage. Therefore, we sought to uncouple the ability of ParB_Bb_ to form foci (due to *parS* binding and spreading on adjacent DNA) from ParB_Bb_ protein levels by constitutively expressing *parB*_*Bb*_*-msfgfp*. In these cells, the fluorescence signal **(S3F Fig)** and protein levels (**S3G Fig)** of ParB_Bb_-msfGFP in AP were stronger than upon expression from the native locus. Still, overproduced ParB_Bb_-msfGFP was diffuse in AP cells **(Fig 3E)**, consistent with our previous results with ParB_Bb_-mCherry [47]. Furthermore, these cells displayed the same phenotypes observed when the untagged ParB_Bb_ is constitutively produced (i.e., morphological and chromosome segregation defects; **S3H, S2B Figs)**, further supporting the relevance of these ParB_Bb_ fusions. Thus, a third level of control is applied to ParB_Bb_ in *B*. *bacteriovorus* to prevent its accumulation at the centromere, regardless of its protein level.

### ParB_Bb_ specifically accumulates on *B*. *bacteriovorus parS* in a CTP-dependent manner

**Fig 4:**
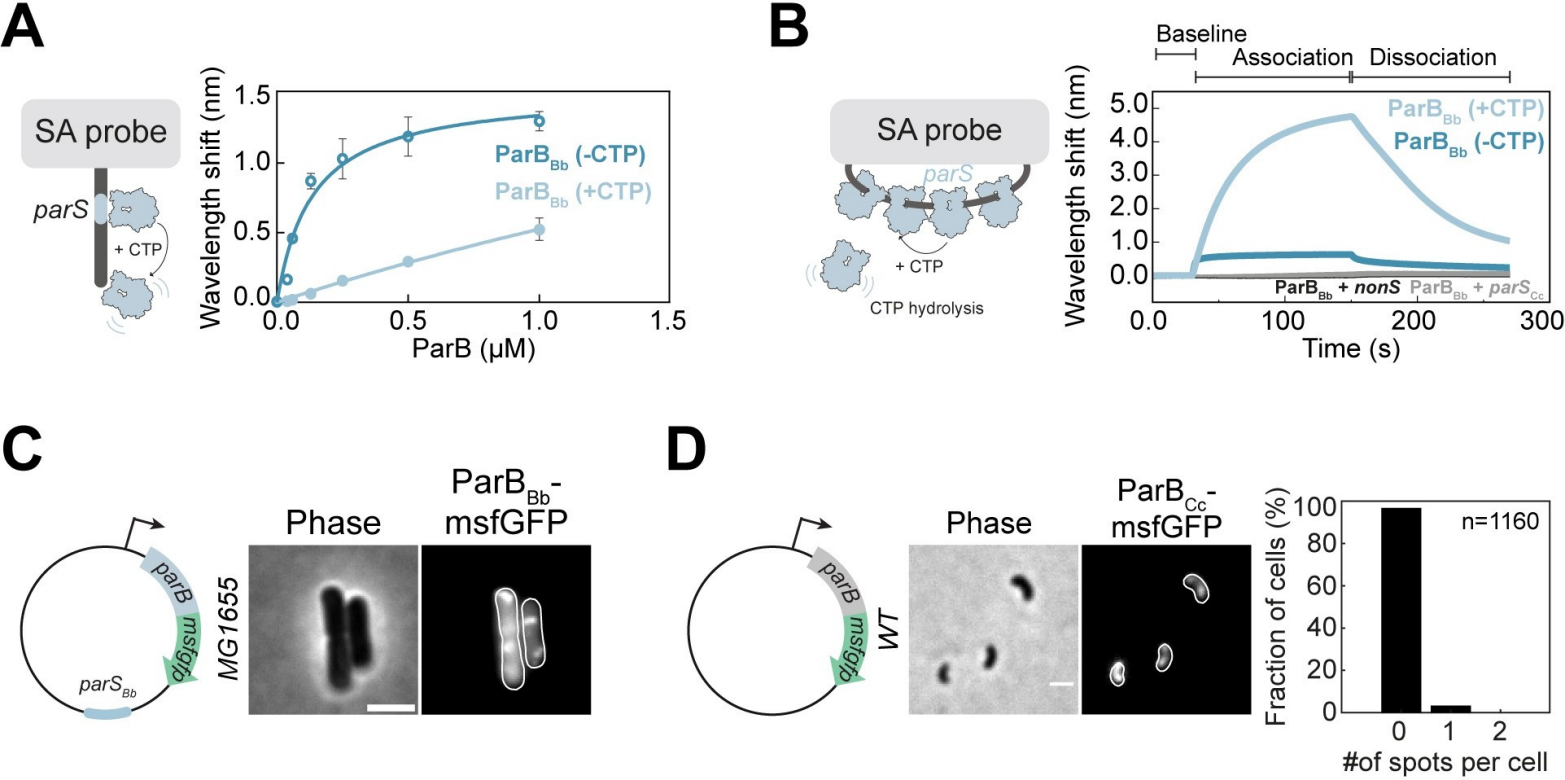
ParB_Bb_ is a CTPase with a specific affinity for its cognate *parS*_*Bb*_. (A) CTP reduces the binding of ParB_Bb_ on *parS*_*Bb*_ on a linear substrate. Left: schematics of the Streptavidin (SA)-coated probe and the linear 40-bp DNA substrate containing the cognate *parS*_*Bb*_ sequence. Right: ParB_Bb_ titration curves in the presence or absence of CTP obtained from biolayer interferometry (BLI) analysis (see B and Material and Methods). All reactions contained 1 mM CTP (when appropriate), 0.5 μM 40 bp *parS*_*Bb*_ DNA, and an increasing concentration of ParB_Bb_. The BLI experiment was done in triplicates; data were fitted to calculate the binding affinity constant Kd (nM); Kd = 134 ± 23 nM for the 40 bp *parS*_*Bb*_, and Kd = 5500 ± 397 nM for the 40 bp *parS*_*Bb*_ + 1mM CTP. In this assay, ParB_Bb_ spreading displaces it from the short linear *parS* fragments by pushing it into the solution. The higher Kd constant (lower *parS* affinity) in the presence of CTP thus indicates CTP-induced spreading of ParB_Bb_. (B) ParB_Bb_ accumulation at *parS*_*Bb*_ is specific and CTP-dependent. Left: schematics of the SA-coated probe and a closed DNA substrate with a *parS* sequence, allowing ParB accumulation through *parS* binding and CTP binding-dependent spreading. CTP hydrolysis removes ParB from the DNA. Right: BLI analysis of the interaction between 1 μM ParB_Bb_-6xhis in the presence or absence of 1 mM CTP and a 180 bp DNA substrate containing the cognate *parS*_*Bb*_ (blue), scrambled *nonS* (black) or non-cognate *parS*_*Cc*_ (grey) sequence. The BLI probe was dipped into a buffer-only solution (base), then into a premix of protein +/- CTP (association), and finally returned to a buffer-only solution (dissociation). Each BLI experiment was done in triplicate, and a representative sensorgram is presented. (C) ParB_Bb_ protein can bind to its cognate *parS* sequence in *E*. *coli*. Representative phase contrast and fluorescence images of *E*. *coli* carrying a plasmid with *parS*_*Bb*_ and allowing constitutive production of ParB_Bb_-msfGFP (GL1669; plasmid schematics on the left). The scale bar is 2 μm. See Fig 5D for histogram representing the percentage of cells with zero, one, or two ParB_Bb_-msfGFP foci in the same strain. (D) A constitutively produced fusion of *Caulobacter crescentus* ParB (ParB_Cc_) to msfGFP does not form foci in AP *B*. *bacteriovorus* cells. Left: representative phase contrast and fluorescence images of AP cells of a WT strain constitutively producing ParB_Cc_-msfGFP from a plasmid (GL2108; schematics on the left). The fluorescence signal shows the partial nucleoid exclusion pattern of freely diffusing cytosolic proteins, characteristic of AP *B*. *bacteriovorus* cells ^1^. Right: histogram of the percentage of cells with zero, one, or two ParB_Cc_-msfGFP foci in the same strain; n indicates the number of cells analyzed in a representative experiment. The scale bar is 1 μm. See also **S4 Fig**.

The formation of partitioning complexes in other species requires both the initial binding and the spreading of ParB on the DNA [8,10,11,13,14,73]. To investigate ParB_Bb_’s ability to perform these two critical functions, we assessed its capacity to bind and spread from *parS* and to hydrolyze CTP *in vitro*. The two *parS* sequences in the proximity of the *B*. *bacteriovorus* chromosomal *oriC* (*parS*_*Bb*_) are identical and closely resemble the *parS* consensus **(S4A Fig)** [2]. In a biolayer interferometry assay, purified ParB_Bb_ bound a linear DNA substrate carrying *parS*_*Bb*_, and the addition of CTP triggered ParB_Bb_ sliding and falling off (marked by the higher Kd in the presence of CTP; **Fig 4A**), like other characterized ParB homologs [11,13–15,73]. Moreover, ParB_Bb_ accumulated on a closed DNA loop containing *parS*_*Bb*_ in a CTP-dependent manner, reflecting the capacity of ParB_Bb_ to bind *parS*_*Bb*_ and escape onto neighboring DNA **(Fig 4B)**. This accumulation was specific to the cognate *parS*_*Bb*_, as no binding was observed when we used a scrambled *parS* sequence (*nonS;* **Fig 4B**) or *parS* from *Caulobacter crescentus* (*parS*_*Cc*_; **Fig 4B**). Finally, measurements of inorganic phosphate release showed that ParB_Bb_ hydrolyses CTP but no other NTPs in the presence of *parS*_*Bb*_ **(S4B Fig)**. Thus, the *B*. *bacteriovorus* ParB protein is a bona-fide *parS*-binding CTPase. Consistent with those *in vitro* data, ParB_Bb_ specifically accumulated on *parS*_*Bb*_ *in vivo* in *E*. *coli* (which lacks an endogenous ParABS), used as a heterologous system. Indeed, ParB_Bb_-msfGFP formed foci when produced in *E*. *coli*, only in the presence of a plasmid carrying *parS*_*Bb*_ and not when the plasmid carried the non-cognate *parS*_*Cc*_ (**Figs 4C and S4C**). Interestingly, ParB from *Caulobacter crescentus* (ParB_Cc_) also failed to form a focus and was diffuse in the cytoplasm when ectopically produced in AP *B*. *bacteriovorus* cells **(Fig 4D)**, even though ParB_Cc_ can bind *parS*_*Bb*_ *in vitro* **(S4D Fig)** and in *E*. *coli* **(S4E Fig)**. Therefore, the absence of ParB_Bb_·*parS*_*Bb*_ complex in non-replicative *B*. *bacteriovorus* cells is unlikely to result from an unusual functionality of the ParB_Bb_ protein itself.

### The *parS*_*Bb*_ chromosomal context prevents ParB_Bb_ accumulation in non-replicative cells

**Fig 5:**
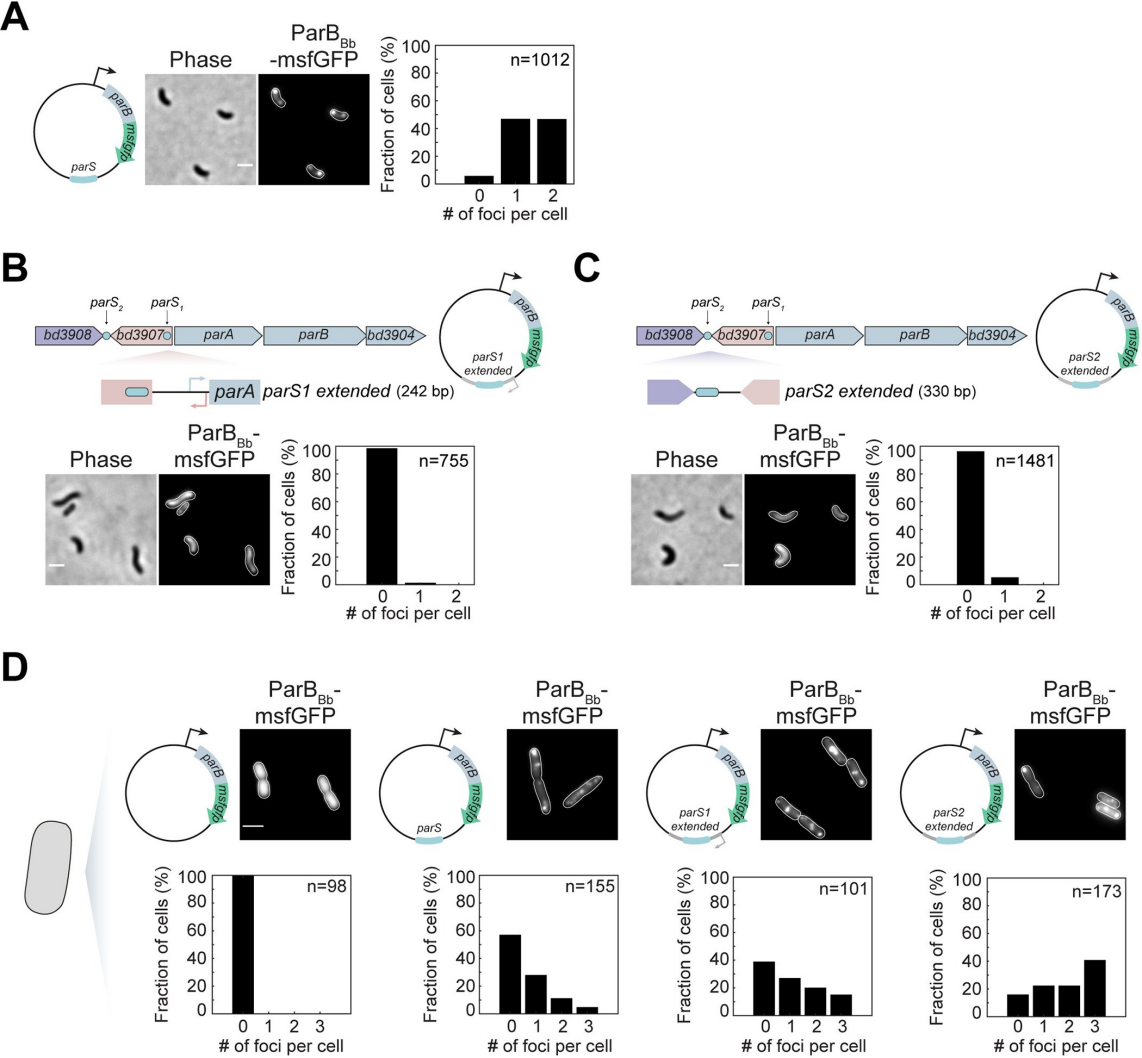
The genomic context of *parS*_*Bb*_ plays a role in ParB_Bb_ localization. (A) Changing the native context of the *parS*_*Bb*_ enables ParB_Bb_ binding. Left: representative phase contrast and fluorescence images of AP cells of WT *B*. *bacteriovorus* strain constitutively producing ParB_Bb_-msfGFP from a plasmid carrying a copy of the *parS*_*Bb*_ sequence (GL1751). Right: histogram representing the percentage of cells with zero, one, or two ParB_Bb_-msfGFP foci in the same strain. (B-C) Short chromosomal regions around each *parS*_*Bb*_ site are enough to prevent ParB_Bb_ from clustering on the centromere. Top: schematic representation of the *parAB* operon in *B*. *bacteriovorus* and the upstream *parS* sequences; chromosomal regions around *parS*_*Bb*_ sequences (“extended *parS”*) cloned in the plasmids are indicated; plasmid schematics are shown on the right. Bottom: representative phase contrast and fluorescence images of AP cells of WT strain constitutively producing ParB_Bb_-msfGFP from a plasmid carrying *parS*_*1extended*_ (B, GL1749) or *parS*_*2extended*_ (C, GL1925); histogram representing the percentage of cells with zero, one, or two ParB_Bb_-msfGFP foci in the same strain. Scale bars are 1 μm. (D) The genomic context of the *parS*_*Bb*_ prevents ParB_Bb_ focus formation only in *B*. *bacteriovorus*. Top: representative phase contrast and fluorescence images of *E*. *coli* strains carrying plasmids as in Figs 3D, 5A–5C, constitutively producing ParB_Bb_-msfGFP in the absence of *parS* (GL1661), or in the presence of *parS*_*Bb*_ (GL1669), *parS*_*1extended*_ (GL1737) or *parS*_*2extended*_ (GL1899), respectively. Schematics illustrate the *parB*_*Bb*_*–msfgfp* expression plasmid with or without (extended) *parS* sequences. Bottom: histogram representing the percentage of cells with zero, one, or two ParB_Bb_-msfGFP foci in the same strains. The scale bar is 1 μm. n indicates the number of cells analyzed in a representative experiment; all experiments were performed at least twice. See also **S5 Fig**.

Having established that ParB_Bb_ activities are not *per se* drastically different than previously characterized ParB homologs, we investigated the possibility that in AP cells, the chromosomal context of the centromere is incompatible with ParB_Bb_ accumulation. Moving one or both *parS*_*Bb*_ sites to a remote location on the chromosome is expected to result in pleiotropic effects due to a major perturbation of segregation, which would prevent unambiguous interpretation. Instead, we addressed whether the genomic context impacts ParB_Bb_ localization by cloning a *parS*_*Bb*_ site on a replicative plasmid and constitutively producing ParB_Bb_-msfGFP from the same vector in *B*. *bacteriovorus* **(Fig 5A)**. Remarkably, we found that ParB_Bb_-msfGFP localizes as clear foci in AP cells only when the plasmid carries the intact *parS*_*Bb*_. No focus was observed when we used a mutated *parS*_Bb_* as a control **(S5A Fig)**. These results indicate that in AP cells, ParB_Bb_ has the capacity to bind *parS*_*Bb*_ specifically but only when *parS*_*Bb*_ is out of its native chromosomal context. Moreover, AP *B*. *bacteriovorus* cells carrying a *parS*_*Bb*_ + *parB*_*Bb*_*-msfgfp*^*++*^ vector did not exhibit the sick phenotype observed in *parB*_*Bb*_^*++*^ cells **(S5B Fig**, compared with **S2C Fig)**, presumably because the plasmidic *parS*_*Bb*_ titrates the excess ParB_Bb_ present in the cytoplasm, thereby alleviating the negative effect associated with its overproduction. Hence, our data show that the chromosomal context is an essential factor underlying the unique subcellular distribution of ParB_Bb_ in *B*. *bacteriovorus*.

To obtain clues on the difference between the chromosomal and plasmidic *parS*_*Bb*_ leading to distinct ParB_Bb_ localization patterns, we tested the impact of the *parS*_*Bb*_ surrounding regions. We constructed two plasmids carrying the *parS*_*Bb*_ sequence flanked by their short upstream and downstream chromosomal regions (named here *parS1 extended* and *parS2 extended*, spanning 242 bp and 330 bp, respectively**, Fig 5B–5C**). Strikingly, the overproduced ParB_Bb_-msfGFP failed to form a fluorescent focus in both cases **(Fig 5B–5C)**. Instead, the signal was diffuse in AP cells, which exhibited the characteristic *parB*_*Bb*_^*++*^ phenotype **(S5B Fig**, compared with **S2C Fig)**. On the contrary, ParB_Bb_ could form a fluorescent focus in the same strains later during growth **(S5D–S5E Fig)**. The effect of the DNA adjacent to *parS* sites was limited to *B*. *bacteriovorus* since in *E*. *coli*, ParB_Bb_-msfGFP localized as foci despite the addition of *parS1* or *parS2 extended* regions **(Fig 5D)**. Therefore, our results suggest that a *Bdellovibrio*-specific factor makes the chromosomal environment of *parS*_*Bb*_ sites incompatible with ParB_Bb_ clustering during the non-proliferative phase of the cell cycle.

## Discussion

**Fig 6:**
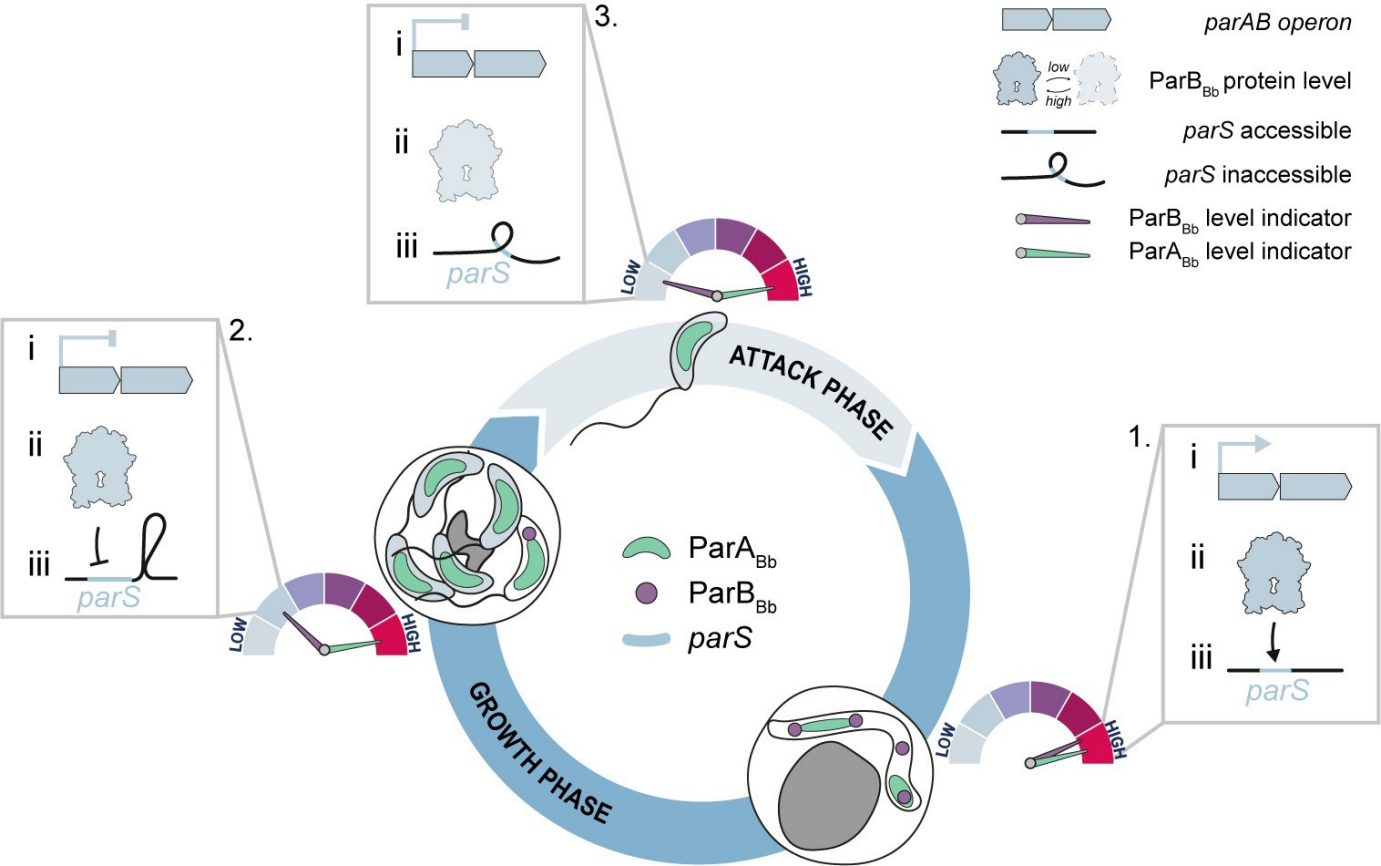
Model for coupling cell cycle progression and centromere organization through multi-layered regulation of the ParABS system in *B*. *bacteriovorus*. Schematic representation of the subcellular localization of ParA_Bb_ (green) and ParB_Bb_ (magenta) throughout the predatory cell cycle in *B*. *bacteriovorus*. The three levels of ParABS regulation are i) transcription of the *parAB* operon, ii) ParA_Bb_ and ParB_Bb_ protein levels, and iii) accessibility of the chromosomal *parS* site. In growing cells (1), the *parAB* operon transcription is "on", ParB_Bb_ and ParA_Bb_ levels are high, and *parS* is accessible, enabling ParB_Bb_ to form nucleoprotein complexes on the centromere. The ParABS system rearranges concomitantly with non-binary division (2), transitioning towards an “off” state. During the attack phase (3), ParA_Bb_ levels remain high, but ParB_Bb_ levels are low due to potential protein degradation at the end of the growth phase, combined with the absence of new transcription of the *parAB* operon. The *parS* chromosomal context is incompatible with the formation of a ParB_Bb_ complex at the centromere during this stage of the cell cycle.

Altogether, our study provides novel insights into the complex regulation of the conserved ParABS system in a bacterium that segregates multiple copies of its chromosome during its non-binary proliferation. We followed the subcellular dynamics, expression, and protein levels of the key players in that system, the proteins ParA and ParB, throughout the predatory cell cycle of *B*. *bacteriovorus*. Additionally, we assessed the ability of ParB_Bb_ to form nucleoprotein complexes on *parS* sites *in vitro* and *in vivo*. We discovered that the ParABS system is coupled to cell cycle progression via three levels of regulation that modulate (i) *parA*_*Bb*_ and *parB*_*Bb*_ gene expression, (ii) ParA_Bb_ and ParB_Bb_ protein levels, and (iii) chromosomal *parS* accessibility during the non-proliferative and replicative stages of the predator lifecycle (**Fig 6**). Furthermore, our data indicate that the combination of these layers of cell cycle-dependent control contributes to clearing the *B*. *bacteriovorus* cell and the centromere from ParB_Bb_ during the non-proliferative stage, which appears crucial for cell cycle progression (see below).

### Intricate subcellular dynamics of ParA_Bb_ and ParB_Bb_ during the replicative stage of the cell cycle

We investigated the localization of ParA_Bb_ in *B*. *bacteriovorus* during the predatory cell cycle using a functional, natively produced fluorescent fusion. The subcellular localization of ParA has been observed in *Streptomyces* species, which also feature a polyploid growth stage [74,75]. While the chromosome is copied multiple times during vegetative growth in these bacteria, the ParABS system only acts later in a distinct developmental stage to separate nucleoids in future spores [76]. Thus, our data provide the first visualization of the ParA protein in a bacterium in which segregating multiple chromosome copies is an integral part of the cell cycle, rather than a conditional process as in *Streptomyces*. In non-replicative cells, ParA_Bb_ is uniformly distributed on the nucleoid, in contrast with the ParA gradient observed in other species in which *oriC* is localized at one cell pole [3]. The absence of a ParB_Bb_·*parS* nucleoprotein complex at the centromere-like region (see below) might explain this non-polarized ParA_Bb_ pattern in AP *B*. *bacteriovorus* cells. Furthermore, our observations of ParA_Bb_ and ParB_Bb_ during chromosome replication and partitioning in the filamentous predator cell revealed several dynamic ParA_Bb_ “clouds” between segregating ParB_Bb_ foci, demonstrating that the ParABS system segregates multiple chromosomal copies simultaneously in the *B*. *bacteriovorus* cell.

In contrast to binary-dividing species like *C*. *crescentus*, the partitioning complexes do not move from one pole to another across the entire predator cell. Instead, ParB_Bb_ foci adopt a clear “beads-on-a-string” pattern ([47] and this study), reminiscent of the ParABS-mediated positioning of low-copy plasmids at non-polar locations [77]. It is still unclear how the segregation of each chromosome is spatially confined within the filamenting *B*. *bacteriovorus* cell body (which is filled with other replicating and segregating copies of the chromosome) to achieve this regular distribution [47]. We found that perturbations in the ParABS system, such as alterations of the ParA_Bb_:ParB_Bb_ balance, prevent this exquisite arrangement of newly synthesized centromeres. In other bacteria, specific hub proteins at the cell poles polarize the choreography of ParABS-dependent segregation [29], but no homologs of these proteins were identified in *B*. *bacteriovorus*. Future research will determine whether this bacterium uses polar landmarks or other mechanisms to spatially organize chromosome partitioning along the mother cell.

### Coupling centromere organization to cell cycle progression

Our previous work uncovered a striking difference between the ParB protein in *B*. *bacteriovorus* and those characterized in other species. Whereas ParB homologs always localize at the *parS* site [7,35,37,55–65], ParB_Bb_ is unable to accumulate at the chromosomal centromere in non-replicative cells, independently of its protein levels [47]. This study offers unambiguous support to this finding by showing that (i) the unique on-off localization pattern of ParB_Bb_ is independent of the fluorescent tag, and (ii) the protein fusions used to monitor ParB_Bb_ subcellular localization and abundance represent reliable reporters of the native protein during the entire *B*. *bacteriovorus* cell cycle. Furthermore, we identified ParB_Bb_ as a bona-fide CTPase like several other ParB homologs [10,13,73,78], which strongly suggests that ParB_Bb_ accumulation at the centromere results from its binding on *parS* and CTP-dependent sliding on adjacent DNA, while subsequent CTP hydrolysis unloads the protein.

Remarkably, we found that the absence of ParB_Bb_·*parS* complex depends on the chromosomal context of *parS*, as ParB_Bb_ formed a focus in *B*. *bacteriovorus* AP cells when *parS* was present on a plasmid. This result indicates that fluctuations in the cellular concentration of CTP, if any, are not responsible for the cell cycle-dependent localization of ParB_Bb_. It also hints that ParB_Bb_ has the capacity to bind and spread from *parS* in the absence of ongoing chromosome replication, even though the first ParB_Bb_ focus systematically appears after DNA replication initiation in GP cells [47]. Moreover, our data reveal that the direct chromosomal context of *parS* sites has a major role in preventing ParB_Bb_ complex formation, as ParB_Bb_ lost its ability to accumulate on the plasmidic *parS* when this sequence was flanked by short DNA stretches adjacent to *parS1* or *parS2* on the chromosome. This intriguing effect was only observed in *B*. *bacteriovorus*, and not in *E*. *coli* cells. Therefore, we propose that the local topology of the DNA in the immediate vicinity of the *parS* sequences, possibly induced by *B*. *bacteriovorus*-specific proteins during the GP-AP transition, might interfere with *parS* binding and/or the spreading of ParB_Bb_. Identifying the specific inhibition that prevents ParB_Bb_ from accumulating at the centromere in a cell-cycle and chromosomal context-dependent manner will certainly reveal additional complexity in bacterial centromere organization.

### Cell cycle-dependent fluctuations of the ParA and ParB protein balance

The ParA_Bb_:ParB_Bb_ balance appears to be crucial for *B*. *bacteriovorus* cell cycle progression, as constitutive production of ParA_Bb_ or ParB_Bb_ throughout the cell cycle led to cellular dysfunction, unless both proteins are overproduced. It is likely that the excess of either protein interferes with their fine-tuned interplay during the chromosome segregation process, resulting in the observed phenotypes. The compensatory effect of the double ParA_Bb_ and ParB_Bb_ overproduction is consistent with this idea.

Intriguingly, our results also hint that ParA_Bb_ and ParB_Bb_ protein levels are differentially controlled in the non-replicative stage of the *B*. *bacteriovorus* cell cycle and suggest that ParB_Bb_ is specifically degraded in a cell cycle-dependent manner. Indeed, although the co-expression of *parA*_*Bb*_ and *parB*_*Bb*_ is turned off during AP, protein concentration is not expected to change between the mother cell and the newborn cells upon cell division. Yet, ParB_Bb_ protein levels dropped within minutes at the GP-AP transition, becoming undetectable in the AP, unlike ParA_Bb_. Whereas there has been no report of a mechanism regulating ParB degradation in other bacteria, a recent study identified ParB as a candidate substrate of the conserved Lon protease in *C*. *crescentus* [79]. Future investigation will be needed to reveal the potential proteolytic mechanism that depletes ParB_Bb_ at the end of the *B*. *bacteriovorus* growth phase.

While it is clear that ParB_Bb_ levels are tightly regulated during the *B*. *bacteriovorus* cell cycle, the physiological role of the disappearance of ParB_Bb_ at the GP-AP transition remains to be determined. We hypothesize that in addition to the importance of proper ParA_Bb_ and ParB_Bb_ levels during growth to ensure accurate chromosome segregation, *B*. *bacteriovorus* benefits from clearing the cell from ParB_Bb_ in the AP. This idea is rationalized by the incapacity of ParB_Bb_ to cluster at the chromosomal *oriC* during that stage, which would result in the entire ParB_Bb_ pool being free in the cytoplasm in the absence of degradation. We propose that such excess of cytosolic ParB_Bb_ in AP cells introduces unknown deleterious effects, since we observed that the ParB_Bb_ overproduction phenotype was largely alleviated when the protein could form a *parS*-bound cluster on a plasmid in AP cells.

Although studies in *Streptomyces coelicolor* have shown developmental stage-specific modulation of ParA and ParB protein levels resulting from the differential control of *parAB* transcription [41], to the best of our knowledge, this is the first report of fluctuating ParA:ParB protein ratios across the lifecycle of a bacterium. Altogether, our study sheds new light on the intricate dynamics and adaptation of the conserved ParABS system in bacteria. Our work hints that investigating the ParABS system in organisms with distinctive lifestyles will unveil new levels of complexity in centromere organization, chromosome segregation, and cell cycle control.

## Materials and methods

### Strains

The strains and plasmids used in this study are listed in **S1 Table**, along with their respective construction methods (**S2 Table**). Standard molecular cloning techniques were employed, and DNA assembly was conducted using the NEBuilder HiFi mix from New England Biolabs. All oligos used in the study can be found in **S3 Table**. The *B*. *bacteriovorus* strains were produced from the wild-type HD100 strain, while the *E*. *coli* strains employed as prey were generated from MG1655. Microscopy, conjugation, bacterial two-hybrid, and POLAR assay were conducted utilizing *E*. *coli* MG1655, S17-λpir, BL21, BTH101, and TOP10, CC118 λpir, TB28, respectively. All plasmids were introduced into *B*. *bacteriovorus* through mating, following the methodology described below. Scarless allelic replacements into the HD100 chromosome were achieved via a two-step recombination approach using a pK18mobsacB-derived suicide vector. The overexpression experiments were performed using the low-copy plasmid pTNV215 (RSF1010 replicon) as a backbone [47]. Chromosomal modifications were screened by PCR and verified by Sanger DNA sequencing, following the protocol detailed in [47].

### Routine culturing of *B*. *bacteriovorus* and *E*. *coli*

*E*. *coli* strains were grown in LB medium. For imaging experiments, overnight starter cultures from single colonies were diluted at least 1:500 in fresh medium until exponential phase. *B*. *bacteriovorus* strains were grown as described in DNB medium (Dilute Nutrient Broth, Becton, Dickinson, and Company) supplemented with 2 mM CaCl_2_ and 3 mM MgCl_2_ salts in the presence of *E*. *coli* prey at 30°C with continuous shaking [80]. When appropriate, antibiotic-resistant *E*. *coli* strains were used as prey for overnight culturing of the corresponding antibiotic-resistant *B*. *bacteriovorus*. When required, kanamycin, gentamycin, ampicillin, chloramphenicol, or tetracycline was added in liquid and solid media at 50 μg/ml, 10 μg/ml, 50 μg/ml, 15 μg/ml, and 7.5 μg/ml respectively.

### Plasmid conjugation by mating

Mating was carried out between the *E*. *coli* S17-λpir donor strain carrying the plasmid to be conjugated and the *B*. *bacteriovorus* receiver strain, following the protocol detailed in [47]. The exponentially growing *E*. *coli* donor strains were harvested and washed twice in DNB medium before resuspending in DNB salts at a 1:10 ratio of the initial volume. This donor suspension was combined with an equal volume of fresh overnight lysate of a receiver HD100 strain. The mating mixture was then incubated for a minimum of 4 hours at 30°C with shaking before being plated on a selective medium using the double-layer technique [80]. Single plaques were isolated, and transconjugants were validated by microscopy (when applicable), PCR, and sequencing.

### RT-PCR

A predator: prey mixture (using a 1:1 volume ratio, which minimizes the excess of free attack phase cells) was prepared and incubated for several hours with shaking at 30°C. RNA samples were collected at different times during synchronous predation to track the expression of *parA*_*Bb*_ *(bd3906)*, *parAB*_*Bb*_ (*bd3905-bd3906*), *parB*_*Bb*_ *(bd3905)* and *dnaK* (*bd1298*) throughout the predatory cycle of *B*. *bacteriovorus* on *E*. *coli* prey. A NucleoSpin RNA kit (MACHEREY-NAGEL) or SV Total RNA Isolation System (Promega) was used to isolate RNA, following the manufacturer’s instructions. An additional TURBO DNase (Invitrogen) treatment was performed for 1 hour at 37°C following manufacturer’s instructions. RNA quality was assessed by measuring the 260/280 nm and 260/230 nm absorbance ratios. Reverse transcription (RT) and PCR were carried out utilizing the QIAGEN OneStep RT-PCR kit with the following thermocycling parameters: 50°C for 30 minutes, 94°C for 15 minutes, followed by 30 cycles of 94°C for 1 minute, 50°C for 1 minute, and 72°C for 1 minute, with a final step of 72°C for 10 minutes.

### Bacterial-two hybrid assay (BACTH)

Interaction between protein pairs was assessed by the adenylate cyclase-based bacterial two-hybrid technique, as detailed in [81]. Briefly, the proteins of interest were merged with the isolated T18 and T25 catalytic domains of the *Bordetella pertussis* adenylate cyclase. The two plasmids producing the fusion proteins were introduced into the BTH101 reporter strain, and co-transformants were incubated on a selective medium overnight at 30°C. A single colony from each co-transformation was inoculated into 400 μl LB medium supplemented with ampicillin (200 μg/ml), kanamycin (50 μg/ml), and IPTG (0.5 mM). After incubation overnight at 30°C, 5 μl of each culture was placed onto LB plates supplemented with ampicillin, kanamycin, IPTG (using the abovementioned concentrations), and X-gal (40 ng/μl) and incubated at 30°C. An interaction assay with pKT25-Zip and pUT18C-Zip, two Zip protein domains, was included as a positive control. The experiments were conducted in triplicate, and representative results are presented.

### PopZ-linked apical recruitment (POLAR) assay

Protein-protein interaction between ParA_Bb_ and ParB_Bb_ was assessed using the POLAR assay, similarly as in [68]. Briefly, proteins of interest were fused to msfGFP-H3H4 or mScarlet to generate the bait and prey protein fusions, respectively. Bait- and prey-encoding plasmids, cloned in *E*.*coli* TOP10 and CC118 λpir, respectively, were introduced in electrocompetent *E*. *coli* TB28 cells containing the temperature-sensitive helper plasmid pAH69. Selection of co-transformants and loss of pAH69 were allowed by overnight incubation on LB plates supplemented with chloramphenicol and tetracycline at 37°C. An overnight starter was prepared by inoculating LB medium supplemented with appropriate antibiotics with a single colony and grown at 37°C. A 6 ml fresh co-culture was prepared in LB medium with antibiotics using a 1:100 dilution and incubated at 37°C. Once OD_600_ reached 0.2, cells were collected by centrifugation (5000 rpm for 2 min) and resuspended in 6 ml 1x M9 minimal medium with salts supplemented with 0.2% Casaminoacids. The 6 ml culture was then split into two 3 ml cultures, one supplemented with 100 μM IPTG for prey protein induction only and the other with both 100 μM IPTG and 0.2% arabinose to induce the expression of the prey protein, PopZ_Cc_, and the bait protein. Snapshots were performed following a 2 h induction at 37°C.

### Live-cell imaging

*B*. *bacteriovorus* were grown overnight with the appropriate *E*. *coli* prey, and antibiotics were used when needed. Subsequently, they were grown on wild-type MG1655 for at least one generation without antibiotics before the beginning of the imaging experiment. For snapshots of fresh AP *B*. *bacteriovorus*, the cells were deposited on 1.2% agarose pads prepared with DNB-salt media. For the time-lapse imaging of synchronous predation cycles, MG1655 *E*. *coli* cells were grown in 2TYE medium to the exponential phase (OD_600_ = 0.4-0.6), harvested at 5000 x *g* at room temperature for 5 minutes, washed twice, and resuspended in DNB medium. *E*. *coli* and *B*. *bacteriovorus* were then combined in a 1:3 to 1:5 volume ratio to allow the majority of prey cells to be infected. This setup ensured infection of all *E*. *coli* cells, which was optimal for time-lapse imaging. The remaining free *Bdellovibrio* cells did not pose any issue in that setup as they neither proliferate nor can re-infect existing bdelloplasts. In all synchronous predation imaging experiments, the prey-predator mixing step was indicated as time 0. The cells were either directly deposited on DNB-agarose pads for imaging or left shaking at 30°C before imaging for the indicated durations. In time-lapse experiments, the same fields of view on the pad were imaged at regular intervals, with the enclosure temperature set to 27°C. When appropriate, prior to imaging, cells were incubated for 5 minutes with DAPI (Life Technologies) at a final concentration of 5 μg/ml for nucleoid staining experiments. Regarding snapshots of *E*. *coli* strains, overnight cultures were diluted at least 1:500 and allowed to grow to the exponential phase before being deposited on 1.2% agarose pads prepared with PBS buffer supplemented with 0.2% glucose, 0.2% casamino acids, 1 μg/ml thiamine, 2 mM MgSO_4_, and 0.1 mM CaCl2), except for POLAR strains which were diluted 1:100 and imaged on 1.2% agarose pads prepared with 1x M9 minimal medium with salts following induction as described above.

### Image acquisition

Phase contrast and fluorescence images were obtained using a Nikon Ti2-E fully-motorized inverted epifluorescence microscope (Nikon) equipped with a CFI Plan Apochromat l DM 100x 1.45/0.13 mm Ph3 oil objective (Nikon), a Sola SEII FISH illuminator (Lumencor), a Prime95B camera (Photometrics), a temperature-controlled light-protected enclosure (Okolab), and filter-cubes for DAPI (32 mm, excitation 377/50, dichroic 409, emission 447/60; Nikon), mCherry (32 mm, excitation 562/40, dichroic 593, emission 640/75; Nikon), and GFP (32 mm, excitation 466/40, dichroic 495, emission 525/50; Nikon). Multi-dimensional image acquisition was supervised using the NIS-Ar software (Nikon). The pixel size was 0.074 μm using the 1.5X built-in zoom lens of the Ti2-E microscope. The same LED illumination and exposure times were applied when capturing images of various strains and/or conditions in one experiment and were set to a minimum for time-lapse acquisitions to limit phototoxicity.

### Image processing

To prepare figures, images were processed using FIJI [82], with contrast and brightness settings being kept consistent for all regions of interest in each figure unless stated otherwise. Denoising (Denoise.ai, Nikon) was applied to all phase contrast and fluorescence channels for **Fig 1C, 1F, 1G** to improve the display of time-lapse images captured with low exposure (necessary to preserve cell viability). Figures were constructed and labeled using Adobe Illustrator. *B*. *bacteriovorus* outlines were obtained with Oufti [83] for AP cells, or drawn manually in Adobe Illustrator for GP cells within bdelloplasts.

### Protein overexpression and purification

Expression and purification of ParB_Bb_ with a C-terminal His-tag were conducted as follows. *E*. *coli* BL21(DE3) cells containing pET21, which expresses C-terminal His-tagged ParB_Bb_, were cultivated at 37°C in autoinducing media supplemented with ampicillin (200 μg/ml). Following a 5-hour induction, cells were harvested by centrifugation and suspended in 35 ml lysis buffer (100 mM Tris-HCl [pH8], 300 mM NaCl, 5% glycerol (v/v), supplemented with a protease inhibitor cocktail (Complete, Roche)). The suspended cells were stored at 20°C. The frozen cells were thawed on ice and lysed using three passages through a French pressure cell at 1500 psi. The suspension was then centrifuged for 15 minutes at 40 000 x *g* at 4°C, and the supernatant was filtered through 0.45 μm filters before loading onto a 1 ml HisTrap column (GE Healthcare) pre-equilibrated with buffer A (100 mM Tris-HCl [pH8], 300 mM NaCl, and 5% [v/v] glycerol). The protein was eluted from the column using an increasing imidazole gradient (15–300 mM) in the same buffer. As a final purification step, size-exclusion chromatography was performed using a HiLoad 16/60 Superdex 75 column (GE Healthcare) with buffer B (100 mM Tris-HCl [pH8], 300 mM NaCl, and 5% [v/v] glycerol). The ParB_Bb_-containing fractions were pooled and analyzed for purity using SDS-PAGE. Glycerol was added to the ParB_Bb_ fractions to a final volume of 10% (v/v), followed by 10 mM EDTA and 1 mM DTT. The purified ParB_Bb_ was aliquoted, snap-frozen in liquid nitrogen, and stored at –80°C.

### Construction of DNA substrates for BLI assays

All DNA constructs were designed using VectorNTI (ThermoFisher) and synthesized chemically (gBlocks dsDNA fragments, IDT). To produce a linear biotinylated 40 bp DNA substrate, complementary oligos (with and without biotin) were heated at 98°C for 5 min before being left to cool down to room temperature overnight to form 50 mM double-stranded DNA. A 180 bp DNA construct was created with M13F and M13R homologous regions at each end. To produce a dual biotin-labeled DNA substrate, PCR reactions were carried out using a 2x GoTaq PCR master mix (Promega), biotin-labeled M13F and biotin-labeled M13R primers, and gBlocks fragments as a template. PCR products were separated by electrophoresis and then purified from the gel.

### Measurement of protein-DNA interaction by bio-layer interferometry (BLI)

Bio-layer interferometry experiments were performed using a BLItz system equipped with Dip-and-Read Streptavidin Biosensors (Molecular Devices) as previously described [15]. The streptavidin biosensor was first equilibrated in a low-salt binding buffer (100 mM Tris-HCl (pH 8), 100 mM NaCl, 1 mM MgCl2, and 0.005% Tween 20) for at least 10 min before each experiment. Biotinylated double-stranded DNA (dsDNA) was then immobilized onto the surface of the biosensor through a cycle of baseline (30 s), association (120 s), and dissociation (120 s). During the association phase, different concentrations of ParB_Bb_ dimers, with or without NTPs at varying concentrations, were added to the binding buffer and allowed to associate with the immobilized DNA for 120 s. Finally, the sensor was transferred into a protein-free binding buffer to monitor the dissociation kinetics for 120 s. The sensor was recycled by dipping in a high-salt buffer (100 mM Tris-HCl (pH 8), 1000 mM NaCl, 1 mM MgCl_2_, and 0.005% Tween 20) for 5 min to remove bound ParB_Bb_. All sensorgrams were recorded and analyzed using the BLItz analysis software (BLItz Pro version 1.2, Molecular Devices) and replotted using DataGraph for presentation. The data were fitted using a one-site-specific binding model in GraphPad Prism Version 5.04 to calculate the binding affinity constant Kd. All experiments were conducted at least in triplicate, and a representative sensorgram is presented in each figure.

### DNA preparation for EnzCheck phosphate assay

A 20 bp palindromic single-stranded DNA fragment (*parS*_*Bb*_: GGATGTTCCACGTGGAACATCC or *parS*_*Cc*_: GGATGTTTCACGTGAAACATCC; 100 mM in 1 mM Tris-HCl pH 8.0, 5 mM NaCl buffer) was heated at 98°C for 5 min before being left to cool down to room temperature overnight to form 50 μM double-stranded DNA. The *parS*_*Bb*_ and *parS*_*Cc*_ sequences are underlined.

### Measurement of NTPase activity by EnzCheck phosphate assay

The EnzCheck Phosphate Assay Kit from Thermo Fisher was used to measure NTP hydrolysis. A reaction buffer containing 1 mM NTP and 1 μM of ParB_Bb_ was analyzed in a Biotek EON plate reader at 25°C 15 hours with readings taken every minute. The reaction buffer consisted of 740 μl of ultrapure water, 50 μl of a 20x customized reaction buffer (100 mM Tris pH 8.0, 2 M NaCl, and 20 mM MgCl_2_), 200 μl of MESG substrate solution, and 10 μL of purine nucleoside phosphorylase (1 unit). Control reactions were performed with buffer only, buffer plus protein, or buffer plus NTP only. The plates were shaken continuously at 280 rpm for 15 hours at 25°C. Each assay was performed at least in triplicate. Data analysis was done using DataGraph, and the NTPase rates were calculated by fitting a linear regression in DataGraph.

### Western blot analysis

For Western blot analysis, the AP samples were prepared following the method used in [47], starting from 1.5 ml cleared predation lysates. For the growth phase samples, a 1:1 volume ratio of *E*. *coli* prey to predator mixture was established, minimizing the excess of attack phase cells. The presence of uninfected, free *E*. *coli* cells did not interfere with downstream Western blot analysis as antibodies recognized proteins present only in *B*. *bacteriovorus*, and normalization by total protein estimates was not done between AP and GP samples (see below). Samples were collected at indicated time intervals for the time course analysis. NuPage Bis-Tris SDS precast polyacrylamide gels (Invitrogen) were used to load the samples and were run at 190 V for 50 minutes in the NuPAGE MOPS SDS running buffer. The assessment of equal total protein loading was carried out using PonceauS staining. The resulting stained membrane was imaged using an Image Quant LAS 500 camera (GE Healthcare). Standard Western blotting procedures were followed using primary antibodies against ParB_Bb_ (rabbit serum obtained after injecting purified ParB_Bb_-6xhis protein; CER Group, Marloie, Belgium), GFP (mouse monoclonal JL-8 antibody, Takara), ParA_Cc_ (rabbit polyclonal antibody, Le lab) and mCherry (polyclonal antibody, Thermo Fisher, product # PA5-34974). Secondary antibodies were goat anti-mouse IgG-peroxidase antibody (Sigma) for JL-8 and goat anti-rabbit IgG-peroxidase antibody (Sigma) for mCherry, ParA_Cc_ and ParB_Bb_. Detection of antibody binding was performed by visualizing chemiluminescence from the reaction of horseradish peroxidase with luminol, imaged with an Image Quant LAS 500 camera (GE Healthcare). The quantification of band intensities was done separately for the AP and GP samples using the ImageQuantTL software. Normalization of band intensities to obtain relative protein levels was done by the total PonceauS intensity in the corresponding lane, used as loading and transfer control. Means and standard deviations were calculated and plotted using GraphPad Prism. Image processing was done with ImageJ. The assembly and annotation of figures were performed using Adobe Illustrator. Replicates of corresponding experiments presented in **Fig 3A, 3C–3D** are provided in **S6A–S6C Fig**, respectively.

### Cell, nucleoid, and spot detection from microscopy images

The automated cell detection tool Oufti [83] was used to detect outlines of AP *B*. *bacteriovorus* cells, uninfected *E*. *coli* cells, or entire bdelloplasts with subpixel precision from phase contrast images. Fluorescence signals were added to cell meshes after background subtraction. Oufti was also used to detect diffraction-limited fluorescent foci and nucleoids with subpixel precision from fluorescence images, using the spotDetection and objectDetection modules. The detected spots and objects were added to the corresponding cell in the Oufti cell lists, including features related to coordinates, morphology, and intensity. The same optimized nucleoid detection parameters were used to ensure consistency and comparisons as done previously [47]. Parameters for spot detection were optimized for each dataset or control set of images.

### Quantitative image analysis from cell meshes

Fluorescence-related analysis, nucleoids, spots-related information, and other properties of individual cells based on microscopy images were extracted from Oufti cellLists data and plotted using custom codes in MATLAB (Mathworks), described below.

### Fluorescence profiles

The custom Matlab script MeanIntProfile.m was used to obtain mean relative fluorescence profiles. Briefly, the fluorescence profile of each cell (corresponding to the array of fluorescence intensity per cell segment provided by the relevant signal field in the Oufti cellList) was first normalized by the corresponding array of *steparea* values (corresponding to the area of each segment of the cell), then divided by their sum to obtain relative fluorescence values for each cell (to account for potential concentration differences between cells). When needed, arrays of relative fluorescence were oriented based on the position of the maximal fluorescence intensity of the indicated signal in each cell half. Cell length vectors were normalized from 0 to 1, and the corresponding relative fluorescence profiles from individual cells were interpolated to a fixed-dimension vector and concatenated before averaging.

### Fluorescence intensity analysis of time-lapse experiments

Bdelloplasts outlines were detected in the first frame of the time-lapse using Oufti and copied to all following frames using MATLAB. The resulting time-lapse cell list was reused in Oufti after background subtraction to add the fluorescence signal(s) to the bdelloplasts. The mean fluorescence profiles per cell over time were computed using a custom Matlab script (MeanFluoIntPerCell_TimeLapse), which extracts and plots the total fluorescence per cell (here bdelloplast) divided by the area of the corresponding cell (bdelloplast) over time.

### Kymographs and demographs

Demographs of relative fluorescence intensity in cells sorted by length were plotted as in [47,84]. When needed, arrays of relative fluorescence intensity values were oriented based on the position of the maximal fluorescence intensity of the indicated signal in each cell half. Kymographs were obtained using the built-in kymograph function in Oufti [83].

### Nucleoid size measurement

To measure nucleoid size, we used the objectDetection module in Oufti [83]. We considered the nucleoid area as a proxy for nucleoid size, based on previous work [85], which demonstrated that variations do not influence nucleoid area measurements in DAPI signal intensity. The nucleoid area was obtained from the nucleoid area field in the Oufti cell lists and exported to MATLAB for all cells with a single nucleoid. The nucleoid area values were then converted to μm^2^ and used to generate violin plots of nucleoid area distributions using R [86].

### Statistical analyses

The sample sizes and the number of repeats are included in the figure legends. Means, standard deviations, and coefficients of variation (CV) were calculated in GraphPad Prism, MATLAB (Mathworks), R, or Microsoft Excel.

### Data, resources, and software availability

Matlab and R codes and datasets have been deposited at Zenodo (doi: 10.5281/zenodo.8325025).

## Supporting information

S1 Fig ParA_Bb_ subcellular localization and interaction with ParB_Bb_. **Related to Fig 1.** (A) The endogenous ParA_Bb_-msfGFP fusion is functional. Violin plots of cell length, cell area, and nucleoid area distributions in *parA*_*Bb*_::*parA*_*Bb*_*-msfgfp* (GL2134) and WT *B*. *bacteriovorus* strains, measured from cells in Fig 1E. The lines indicate the 25, 50, and 75 percent quantiles from bottom to top. Mean, standard deviation and coefficient of variation (CV) values are shown on top of the corresponding plot. n indicate the number of cells analyzed in a representative experiment. (B) ParA_Bb_ is not oriented towards any cell pole in AP cells. Left to right: representative phase contrast and fluorescence images of AP cells of *parA*_*Bb*_::*parA*_*Bb*_*-msfgfp romR*::*romR-mcherry* strain (GL2155); demographs of the corresponding fluorescent signals in the same cells sorted by length and oriented based on RomR-mCherry signal intensity; heatmaps represent relative fluorescence intensities; mean pole-to-pole profiles of relative fluorescence intensity of the corresponding fusions in the same cells. Scale bar is 1 μm. (C) ParA_Bb_ and ParB_Bb_ interact in a bacterial two-hybrid assay. BTH101 reporter cells producing the indicated proteins fused to the T18 or T25 adenylate cyclase domain were spotted on X-gal agar plates supplemented with IPTG. Interaction between two proteins results in blue colony color. The Zip-Zip interaction serves as a positive control. (D) Schematic representation of the POLAR assay. POLAR takes advantage of PopZ from *C*. *crescentus* (grey), which spontaneously forms clusters at the cell poles or septa when produced in *E*. *coli*. The unlabeled PopZ is produced along with an msfGFP-H3H4 fusion to the bait protein of choice (here ParB_Bb_), depicted in magenta; H3H4 is a PopZ self-interacting domain (shown in blue) that is sufficient to draw the bait protein to PopZ clusters. The prey protein (ParA_Bb_) is depicted in green. The prey protein is recruited to the PopZ-bait cluster if prey and bait proteins interact (upper half of the cell), whereas a lack of interaction leaves the localization of the prey protein unmodified by the bait protein (lower part of the cell). (E) ParA_Bb_ does not interact with msfGFP-H3H4 (control). Representative fluorescence images of *E*. *coli* cells expressing ParA_Bb_-mScarlet in the absence (left) or presence (right) of msfGFP-H3H4 without bait fusion. ParA_Bb_-mScarlet (expressed from GL2295*)* displays a nucleoid-bound pattern and is not recruited to the polar PopZ cluster via msfGFP-H3H4; pole-to-pole profiles of relative fluorescence intensity of the corresponding fusions in the one cell. (F) ParA_Bb_ and ParB_Bb_ interact in the POLAR assay. Same as in (E) for cells producing ParA_Bb_-mScarlet as prey (green) in the absence (left) or presence (right) of ParB_Bb_-msfGFP as bait fused to msfGFP-H3H4 (magenta). ParA_Bb_ partially colocalizes with the ParB_Bb_-msfGFP-H3H4 cluster (expressed from GL2294) visible as fluorescent foci (arrowheads); pole-to-pole profiles of relative fluorescence intensity of the corresponding fusions in the same cell. Scale bar is 2 μm. (G) A strain in which both native ParA_Bb_ and ParB_Bb_ are labeled displays morphology and chromosome aspect ratio similar to wild-type. Violin plots of cell length, cell area, and nucleoid area distributions in *parA*_*Bb*_::*parA*_*Bb*_*-msfgfp parB*_*Bb*_::*parB*_*Bb*_*-mcherry* (GL2154) and WT *B*. *bacteriovorus* strains, measured from cells in Fig 1G. The lines indicate the 25, 50, and 75 percent quantiles from bottom to top. Mean and standard deviation values are shown on top of the corresponding plot. n indicate the number of cells analyzed in a representative experiment. All experiments were performed at least twice.
(TIF)

S2 Fig Biphasic expression of the *parA*_*Bb*_ and *parB*_*Bb*_ genes and proper balancing of *parAB*_*Bb*_ expression contributes to progressive *ori* segregation. **Related to Fig 2.** (A) Expression of the *parA*_*Bb*_ and *parB*_*Bb*_ genes, individually, follow the same biphasic pattern as the fragment covering both genes. RT-PCR experiment as in Fig 2A using primer pairs hybridizing within the *parA*_*Bb*_ (left) or the *parB*_*Bb*_ gene (right). (B) Overexpression of *parA*_*Bb*_ leads to chromosome segregation defects. *B*. *bacteriovorus* strain *parB*_*Bb*_::*parB*_*Bb*_*-mcherry /* pTNV215-*parA*_*Bb*_ (GL2129) was mixed with prey and imaged in time-lapse after 120 min with 8-min intervals. Left: phase contrast and fluorescence images of selected time points; arrowhead points to an altered ParB_Bb_-mCherry behavior (patches instead of well-separated foci) during the cell cycle. (C) Overexpression of *parB*_*Bb*_ or *parA*_*Bb*_ leads to pronounced phenotypes, largely rescued by overexpression of both. Violin plots of cell length, cell area, and nucleoid area distributions in WT */* pTNV215-*parB*_*Bb*_ (*parB*_*Bb*_^*++*^, GL1261); WT */* pTNV215-*parA*_*Bb*_ (*parA*_*Bb*_^*++*^, GL1460); WT */* pTNV215-*parB*_*Bb*_-*parA*_*Bb*_ (*parAB*_*Bb*_^*++*^, GL1004) and WT *B*. *bacteriovorus* from cells in Fig 2B. The lines indicate the 25, 50, and 75 percent quantiles from bottom to top. Mean, standard deviation and coefficient of variation (CV) values are shown on top of the corresponding plot. n indicate the number of cells analyzed in a representative experiment. (D) Overexpression of both *parA*_*Bb*_ and *parB*_*Bb*_ has no obvious effect on *ori* segregation. *B*. *bacteriovorus* strain WT */* pTNV215-*parB*_*Bb*_*-mcherry*_*stop*_*-RBS-parA*_*Bb*_*-msfgfp* (GL1004) was mixed with prey and imaged in time-lapse after 75 min with 8-min intervals. Left: phase contrast and fluorescence images of selected time points; arrowheads point to well-distributed ParB_Bb_-mCherry foci (which mark chromosomal *ori*) during the cell cycle; ParA_Bb_-msfGFP not shown for simplicity. All experiments were performed at least twice. Scale bars are 1 μm.
(TIF)

S3 Fig The levels of ParB_Bb_ and ParA_Bb_ vary differently during the cell cycle, and ParB_Bb_ is unable to form foci when overproduced. **Related to Fig 3.** (A) The anti-ParB_Bb_ antibody detects overproduced (right) but not native levels (right) of untagged ParB_Bb_ in AP; control experiment for Fig 3A. Western blots of whole-cell protein extracts from AP cells of *WT B*. *bacteriovorus* and a strain constitutively producing the untagged ParB_Bb_ (GL1261) were probed with an anti-ParB_Bb_ antibody represented in the schematics on the left. (B) Same as in Fig 3B for the strain natively producing ParB_Bb_-msfGFP (GL1654). (C) The protein levels of natively produced ParB_Bb_-msfGFP or ParB_Bb_-mCherry detected with the same anti-ParB_Bb_ antibody compare with the endogenous untagged ParB_Bb_ profile during a synchronized *B*. *bacteriovorus* cell cycle. Western blots of whole-cell protein extracts from *B*. *bacteriovorus* strains *parB*_*Bb*_::*parB*_*Bb*_*-msfgfp* (GL1654) and *parB*_*Bb*_::*parB*_*Bb*_*-mcherry* (GL906) were probed with an anti-ParB_Bb_ antibody, as represented in the schematics. Protein samples are isolated at time points throughout the predatory cell cycle: AP, 1h, 2h, 3h, and 4h after mixing with prey. Arrowhead indicates detected ParB_Bb_ protein during the growth phase. Ponceau staining of the same membranes (where bands were most visible, ~30-50 kDa) is illustrated below each blot as a loading control. Molecular weight markers (kDa) are shown on the side. (D) ParA_Bb_ protein is detected in the AP. Western blots of whole-cell protein extracts from *Caulobacter crescentus* (positive control), *E*. *coli MG1655* (negative control), and *WT B*. *bacteriovorus* (in triplicates), were probed with an anti-ParA_Cc_ antibody, as represented in the schematics on the left. Untagged ParA_Bb_ (~29 kDa) is detected in the AP in all three samples. Ponceau staining of the same membranes (where bands were most visible, ~30-50 kDa) is illustrated below each blot as a loading control. Molecular weight markers (kDa) are shown on the side. (E) Same as in Fig 3B for the strain natively producing ParA_Bb_-msfGFP (GL2134). (F) Mean msfGFP fluorescence measured for AP cells of WT, *parB*_*Bb*_::*parB*_*Bb*_*-msfgfp* (*parB*_*Bb*_*-msfgfp*, GL1654) and *WT /* pTNV215-*parB*_*Bb*_*-msfgfp* (*parB*_*Bb*_*-msfgfp*^*++*^, GL1003). n indicate the number of cells analyzed in a representative experiment; mean values are represented. Error bars indicate standard deviations. (G) Western blots of whole-cell protein extracts from AP cells of WT *B*. *bacteriovorus parB*_*Bb*_::*parB*_*Bb*_*-msfgfp* (*parB*_*Bb*_*-msfgfp*, GL1654, left) and *WT /* pTNV215-*parB*_*Bb*_*-msfgfp* (*parB*_*Bb*_*-msfgfp*^*++*^, GL1003, right) strains were probed with an anti-msfGFP antibody, represented in the schematics on the left. Arrowhead points to the ParB_Bb_-msfGFP protein detected in AP when overproduced. (H) Overproduction of ParB_Bb_-msfGFP leads to morphological and chromosome segregation defects. Violin plots of cell length and nucleoid area distributions for the cells in Fig 3E and WT *B*. *bacteriovorus*. The lines indicate the 25, 50, and 75 percent quantiles from bottom to top. Mean, standard deviation and coefficient of variation (CV) values are shown on top of the corresponding plot. n indicate the number of cells analyzed in a representative experiment. All experiments were performed at least twice.
(TIF)

S4 Fig Investigation of the ParB_Bb_ features near the CTP binding pocket and their impact on *parS*_*Bb*_ recognition. **Related to Fig 4.** (A) *parS*_*Bb*_ sequence matches with the consensus. Top: *parS* sequence from *B*. *bacteriovorus*. Bottom: a sequence logo generated by WebLogo 3.0 [1], using *parS* sequence alignments from [5] as input. (B) ParB_Bb_ is a CTPase. Continuous monitoring of the released inorganic phosphate (Pi) by recording the absorbance at 360 nm overtime at 25°C. The NTP hydrolysis of ParB_Bb_ was also monitored in the presence of ATP, GTP, or UTP, with a 22 bp *parS*_*Bb*_ DNA duplex. (C) ParB_Bb_ does not cluster without *parS* or on non-cognate *parS*. Left: representative phase contrast and fluorescence images of MG1655 *E*. *coli* strain constitutively producing ParB_Bb_-msfGFP from a plasmid carrying no *parS* sequence (GL1661) or the *C*. *crescentus parS* (*parS*_*Cc*_; GL2024). Right: histograms representing the percentage of cells with zero, one, or two ParB_Bb_-msfGFP foci in the same strains. The scale bar is 2 μm; schematics illustrate *parB*_*Bb*_-*msfgfp* expression plasmids. (D) ParB from *C*. *crescentus* (ParB_Cc_) is more promiscuous to *parS* binding than ParB_Bb_. BLI analysis of the interaction between 1 μM ParB_Cc_ and a 40 bp cognate *parS*_*Cc*_ (grey) or a non-cognate *parS*_*Bb*_ (blue). ParB_Cc_ binds both *parS* sequences. (E) ParB_Cc_ can bind to its cognate *parS*_*Cc*_ and a non-cognate *parS*_*Bb*_ in *E*. *coli*. Left: representative phase contrast and fluorescence images of MG1655 *E*. *coli* strain constitutively producing ParB_Cc_-msfGFP from a plasmid carrying *parS*_*Cc*_ (left, GL2025) or *parS*_*Bb*_ (right, GL2026); fluorescent foci are observed in both cases. The scale bar is 2 μm; schematics illustrate the *parB*_*Bb*_-*msfgfp* expression plasmid.
(PDF)

S5 Fig The binding of ParB_Bb_ on plasmidic *parS*_*Bb*_ is specific; titration of the free ParB_Bb_ by *parS*_*Bb*_ rescues the *parB* overexpression phenotype. **Related to Fig 5.** (A) From left to right: representative phase contrast and fluorescence images of AP cells of WT *B*. *bacteriovorus* constitutively producing ParB_Bb_-msfGFP from a plasmid carrying a mutated –*parS*_*Bb*_ (*parS*_Bb_*, GL1541). Right: histogram representing the percentage of cells with zero, one, or two ParB_Bb_-msfGFP foci in the same strain. (B) Only the cytosolic excess ParB_Bb_ is toxic for *B*. *bacteriovorus* AP cells. Violin plots of cell length for the cells in Fig 5A–5C and WT *B*. *bacteriovorus*. The lines indicate the 25, 50, and 75 percent quantiles from bottom to top. Mean, standard deviation, and coefficient of variation (CV) values are shown on top of the corresponding plot. n indicate the number of cells analyzed in a representative experiment; all experiments were performed at least twice. (C) Diffuse ParB_Bb_-msfGFP signal in AP cells carrying plasmidic *parSextended* sequences is not due to protein instability. Western blots of whole-cell protein extracts from AP cells of *B*. *bacteriovorus* WT / pTNV215-*parB*_*Bb*_*-msfgfp*-*parS* (GL1750, AP, fluorescent focus), WT / pTNV215-*parB*_*Bb*_*-msfgfp-parS1extended* (GL1749, AP, diffuse signal), WT / pTNV215-*parB*_*Bb*_*-msfgfp-parS1extended* (GL1925, AP, diffuse signal), WT / pTNV215-*parB*_*Bb*_*-msfgfp* (GL1003, AP, diffuse signal) and WT / pTNV215-*msfgfp* (GL1208, control) strains were probed with an anti-msfGFP antibody, as represented in the schematics on the left. (D-E) ParB_Bb_-msfGFP is able to form foci in the growth phase in strains carrying plasmidic *parSextended* sequences. Time-course experiment with strains (D) GL1749 (WT / pTNV215-*parB*_*Bb*_*-msfgfp-parS1extended*) and (E) GL1825 (WT / pTNV215-*parB*_*Bb*_*-msfgfp-parS2extended*). Cells were mixed with prey and imaged every hour. Phase contrast and fluorescence images of selected timepoints are shown; the white arrowheads indicate the appearance of ParB_Bb_-msfGFP foci.
(TIF)

S6 Fig Replicates and total protein loading control for the Western Blots shown in Fig 3. (A) Top: membranes used in Fig 3A stained with PonceauS, in triplicates. Bottom: Western Blot signal from anti-ParB_Bb_ antibody is shown in triplicates. (B) Same as in (A) for samples used in Fig 3C. (C) Same as in (A) for samples used in Fig 3D. In all cases, PonceauS staining indicates that comparable amounts of total protein were loaded in each set of samples. Unprocessed images and full lanes of PonceauS staining are used for ImageQuantTL quantification, as a proxy for total loaded proteins in order to normalize Western blot band intensities (see Methods).
(TIF)

S1 Table Strain information: *Bdellovibrio bacteriovorus*, *Caulobacter crescentus*, and *E*. *coli* strains used in this study. (DOCX)

S2 Table Construction of *Bdellovibrio bacteriovorus* strains and plasmids used in this study. *pTNV215 is a low-copy plasmid (broad host range RSF1010 replicon).
(DOCX)

S3 Table Oligos used in this study. Overlapping sequences used for cloning by DNA assembly and site-directed mutations are highlighted in black.
(DOCX)

S1 Video Cell-cycle-dependent localization of ParB_Bb_. *B*. *bacteriovorus* strain *parB*_*Bb*_::*parB*_*Bb*_*-msfgfp* strain. (AVI)

S2 Video Cell-cycle-dependent localization of ParA_Bb_. *B*. *bacteriovorus* strain *parA*_*Bb*_::*parA*_*Bb*_*-msfgfp* strain. (AVI)

S3 Video Cell-cycle-dependent localization of ParA_Bb_. *B*. *bacteriovorus* strain *parB*_*Bb*_::*parB*_*Bb*_*-mcherry parA*_*Bb*_::*parA*_*Bb*_*-msfgfp* strain. (AVI)

## References

[1] Tromer EC, Hooff JJE van, Kops GJPL, Snel B. Mosaic origin of the eukaryotic kinetochore. Proc National Acad Sci. 2019;116: 12873–12882. doi: 10.1073/pnas.1821945116 31127038 PMC6601020

[2] Livny J, Yamaichi Y, Waldor MK. Distribution of centromere-like parS sites in bacteria: insights from comparative genomics. J Bacteriol. 2007;189: 8693–8703. doi: 10.1128/JB.01239-07 17905987 PMC2168934

[3] Jalal ASB, Le TBK. Bacterial chromosome segregation by the ParABS system. Open Biology. 2020;10: 200097. doi: 10.1098/rsob.200097 32543349 PMC7333895

[4] Breier AM, Grossman AD. Whole-genome analysis of the chromosome partitioning and sporulation protein Spo0J (ParB) reveals spreading and origin-distal sites on the Bacillus subtilis chromosome. Mol Microbiol. 2007;64: 703–718. doi: 10.1111/j.1365-2958.2007.05690.x 17462018

[5] Funnell BE. The P1 plasmid partition complex at parS. The influence of Escherichia coli integration host factor and of substrate topology. J Biol Chem. 1991;266: 14328–14337. doi: 10.1016/s0021-9258(18)98688-6 1860842

[6] Lin DC-H, Grossman AD. Identification and Characterization of a Bacterial Chromosome Partitioning Site. Cell. 1998;92: 675–685. doi: 10.1016/s0092-8674(00)81135-6 9506522

[7] Lee M-J, Liu C-H, Wang S-Y, Huang C-T, Huang H. Characterization of the Soj/Spo0J chromosome segregation proteins and identification of putative parS sequences in Helicobacter pylori. Biochem Bioph Res Co. 2006;342: 744–750. doi: 10.1016/j.bbrc.2006.01.173 16494844

[8] Tran NT, Stevenson CE, Som NF, Thanapipatsiri A, Jalal ASB, Le TBK. Permissive zones for the centromere-binding protein ParB on the Caulobacter crescentus chromosome. Nucleic Acids Res. 2018;46: 1196–1209. doi: 10.1093/nar/gkx1192 29186514 PMC5815017

[9] Murray H, Ferreira H, Errington J. The bacterial chromosome segregation protein Spo0J spreads along DNA from parS nucleation sites. Mol Microbiol. 2006;61: 1352–1361. doi: 10.1111/j.1365-2958.2006.05316.x 16925562

[10] Osorio-Valeriano M, Altegoer F, Steinchen W, Urban S, Liu Y, Bange G, et al. ParB-type DNA Segregation Proteins Are CTP-Dependent Molecular Switches. Cell. 2019;179: 1512–1524.e15. doi: 10.1016/j.cell.2019.11.015 31835030

[11] Osorio-Valeriano M, Altegoer F, Das CK, Steinchen W, Panis G, Connolley L, et al. The CTPase activity of ParB determines the size and dynamics of prokaryotic DNA partition complexes. Mol Cell. 2021;81: 3992–4007.e10. doi: 10.1016/j.molcel.2021.09.004 34562373

[12] Tišma M, Panoukidou M, Antar H, Soh Y-M, Barth R, Pradhan B, et al. ParB proteins can bypass DNA-bound roadblocks via dimer-dimer recruitment. Sci Adv. 2022;8: eabn3299. doi: 10.1126/sciadv.abn3299 35767606 PMC9242446

[13] Soh YM, Davidson IF, Zamuner S, Basquin J, Bock FP, Taschner M, et al. Self-organization of parS centromeres by the ParB CTP hydrolase. Science. 2019;366: 1129–1133. doi: 10.1126/science.aay3965 31649139 PMC6927813

[14] Antar H, Soh YM, Zamuner S, Bock FP, Anchimiuk A, Rios PDL, et al. Relief of ParB autoinhibition by parS DNA catalysis and recycling of ParB by CTP hydrolysis promote bacterial centromere assembly. Sci Adv. 2021. doi: 10.1126/sciadv.abj2854 34613769 PMC8494293

[15] Jalal AS, Tran NT, Stevenson CE, Chimthanawala A, Badrinarayanan A, Lawson DM, et al. A CTP-dependent gating mechanism enables ParB spreading on DNA. Elife. 2021;10. doi: 10.7554/eLife.69676 34397383 PMC8367383

[16] Leonard TA, Butler PJ, Löwe J. Bacterial chromosome segregation: structure and DNA binding of the Soj dimer--a conserved biological switch. EMBO J. 2005;24: 270–282. doi: 10.1038/sj.emboj.7600530 15635448 PMC545817

[17] Marston AL, Errington J. Dynamic Movement of the ParA-like Soj Protein of B. subtilis and Its Dual Role in Nucleoid Organization and Developmental Regulation. Mol Cell. 1999;4: 673–682. doi: 10.1016/s1097-2765(00)80378-0 10619015

[18] Lim HC, Surovtsev IV, Beltran BG, Huang F, Bewersdorf J, Jacobs-Wagner C. Evidence for a DNA-relay mechanism in ParABS-mediated chromosome segregation. Elife. 2014;3: e02758. doi: 10.7554/eLife.02758 24859756 PMC4067530

[19] Surovtsev IV, Campos M, Jacobs-Wagner C. DNA-relay mechanism is sufficient to explain ParA-dependent intracellular transport and patterning of single and multiple cargos. Proc Natl Acad Sci USA. 2016;113: 201616118–E7276. doi: 10.1073/pnas.1616118113 27799522 PMC5135302

[20] Vecchiarelli AG, Seol Y, Neuman KC, Mizuuchi K. A moving ParA gradient on the nucleoid directs subcellular cargo transport via a chemophoresis force. Bioarchitecture. 2014;4: 154–159. doi: 10.4161/19490992.2014.987581 25759913 PMC4914017

[21] Hu L, Vecchiarelli AG, Mizuuchi K, Neuman KC, Liu J. Directed and persistent movement arises from mechanochemistry of the ParA/ParB system. Proc Natl Acad Sci USA. 2015;112: E7055–64. doi: 10.1073/pnas.1505147112 26647183 PMC4697391

[22] Ebersbach G, Briegel A, Jensen GJ, Jacobs-Wagner C. A self-associating protein critical for chromosome attachment, division, and polar organization in caulobacter. Cell. 2008;134: 956–968. doi: 10.1016/j.cell.2008.07.016 18805089 PMC2614312

[23] Bowman GR, Comolli LR, Zhu J, Eckart M, Koenig M, Downing KH, et al. A polymeric protein anchors the chromosomal origin/ParB complex at a bacterial cell pole. Cell. 2008;134: 945–955. doi: 10.1016/j.cell.2008.07.015 18805088 PMC2745220

[24] Yamaichi Y, Bruckner R, Ringgaard S, Moll A, Cameron DE, Briegel A, et al. A multidomain hub anchors the chromosome segregation and chemotactic machinery to the bacterial pole. Genes Dev. 2012;26: 2348–2360. doi: 10.1101/gad.199869.112 23070816 PMC3475806

[25] Thomaides HB, Freeman M, Karoui ME, Errington J. Division site selection protein DivIVA of Bacillus subtilis has a second distinct function in chromosome segregation during sporulation. Genes Dev. 2001;15: 1662–1673. doi: 10.1101/gad.197501 11445541 PMC312724

[26] Schofield WB, Lim HC, Jacobs-Wagner C. Cell cycle coordination and regulation of bacterial chromosome segregation dynamics by polarly localized proteins. EMBO J. 2010;29: 3068–3081. doi: 10.1038/emboj.2010.207 20802464 PMC2944072

[27] Laloux G, Jacobs-Wagner C. Spatiotemporal control of PopZ localization through cell cycle-coupled multimerization. J Cell Biol. 2013;201: 827–841. doi: 10.1083/jcb.201303036 23751494 PMC3678156

[28] Lin L, Valeriano MO, Harms A, Søgaard-Andersen L, Thanbichler M. Bactofilin-mediated organization of the ParABS chromosome segregation system in Myxococcus xanthus. Nat Comms. 2017;8: 1817. doi: 10.1038/s41467-017-02015-z 29180656 PMC5703909

[29] Laloux G, Jacobs-Wagner C. How do bacteria localize proteins to the cell pole? J Cell Sci. 2014;127: 11–19. doi: 10.1242/jcs.138628 24345373 PMC3874780

[30] Thanbichler M, Shapiro L. MipZ, a Spatial Regulator Coordinating Chromosome Segregation with Cell Division in Caulobacter. Cell. 2006;126: 147–162. doi: 10.1016/j.cell.2006.05.038 16839883

[31] Bigot S, Sivanathan V, Possoz C, Barre F-X, Cornet F. FtsK, a literate chromosome segregation machine. Mol Microbiol. 2007;64: 1434–1441. doi: 10.1111/j.1365-2958.2007.05755.x 17511809

[32] Espéli O, Borne R, Dupaigne P, Thiel A, Gigant E, Mercier R, et al. A MatP-divisome interaction coordinates chromosome segregation with cell division in E. coli. EMBO J. 2012;31: 3198–3211. doi: 10.1038/emboj.2012.128 22580828 PMC3400007

[33] Reyes-Lamothe R, Sherratt DJ. The bacterial cell cycle, chromosome inheritance and cell growth. Nat Rev Micro. 2019;15: 1. doi: 10.1038/s41579-019-0212-7 31164753

[34] Mohl DA, Gober JW. Cell Cycle–Dependent Polar Localization of Chromosome Partitioning Proteins in Caulobacter crescentus. Cell. 1997;88: 675–684. doi: 10.1016/s0092-8674(00)81910-8 9054507

[35] Jung A, Raßbach A, Pulpetta RL, Teeseling MCF van, Heinrich K, Sobetzko P, et al. Two-step chromosome segregation in the stalked budding bacterium Hyphomonas neptunium. Nat Comms. 2019;10: 3290. doi: 10.1038/s41467-019-11242-5 31337764 PMC6650430

[36] Sternon J-F, Godessart P, Freitas RG de, Henst MV der, Poncin K, Francis N, et al. Transposon Sequencing of Brucella abortus Uncovers Essential Genes for Growth In Vitro and Inside Macrophages. Infect Immun. 2018;86. doi: 10.1128/IAI.00312-18 29844240 PMC6056880

[37] Iniesta AA. ParABS System in Chromosome Partitioning in the Bacterium Myxococcus xanthus. Driks A, editor. PLoS ONE. 2014;9: e86897. doi: 10.1371/journal.pone.0086897 24466283 PMC3899335

[38] McLean TC, Le TB. CTP switches in ParABS-mediated bacterial chromosome segregation and beyond. Curr Opin Microbiol. 2023;73: 102289. doi: 10.1016/j.mib.2023.102289 36871427

[39] Pióro M, Jakimowicz D. Chromosome Segregation Proteins as Coordinators of Cell Cycle in Response to Environmental Conditions. Front Microbiol. 2020;11: 588. doi: 10.3389/fmicb.2020.00588 32351468 PMC7174722

[40] Quisel JD, Lin DC-H, Grossman AD. Control of Development by Altered Localization of a Transcription Factor in B. subtilis. Mol Cell. 1999;4: 665–672. doi: 10.1016/s1097-2765(00)80377-9 10619014

[41] Jakimowicz D, Mouz S, Zakrzewska-Czerwinska J, Chater KF. Developmental control of a parAB promoter leads to formation of sporulation-associated ParB complexes in Streptomyces coelicolor. J Bacteriol. 2006;188: 1710–20. doi: 10.1128/JB.188.5.1710-1720.2006 16484182 PMC1426544

[42] Eswara PJ, Ramamurthi KS. Bacterial Cell Division: Nonmodels Poised to Take the Spotlight. Annu Rev Microbiol. 2017;71: annurev-micro-102215-095657. doi: 10.1146/annurev-micro-102215-095657 28697666 PMC6291244

[43] Kysela DT, Randich AM, Caccamo PD, Brun YV. Diversity Takes Shape: Understanding the Mechanistic and Adaptive Basis of Bacterial Morphology. PLoS Biol. 2016;14: e1002565. doi: 10.1371/journal.pbio.1002565 27695035 PMC5047622

[44] Angert ER. Alternatives to binary fission in bacteria. Nat Rev Microbiol. 2005;3: 214–224. doi: 10.1038/nrmicro1096 15738949

[45] Rotem O, Pasternak Z, Jurkevitch E. The Prokaryotes, Deltaproteobacteria and Epsilonproteobacteria. 2014; 3–17. doi: 10.1007/978-3-642-39044-9_379

[46] Laloux G. Shedding Light on the Cell Biology of the Predatory Bacterium Bdellovibrio bacteriovorus. Front Microbiol. 2020;10: 3136. doi: 10.3389/fmicb.2019.03136 32038570 PMC6985089

[47] Kaljević J, Saaki TNV, Govers SK, Remy O, Raaphorst R van, Lamot T, et al. Chromosome choreography during the non-binary cell cycle of a predatory bacterium. Curr Biol. 2021;31: 3707–3720.e5. doi: 10.1016/j.cub.2021.06.024 34256020 PMC8445325

[48] Abram D, Melo JC e, Chou D. Penetration of Bdellovibrio bacteriovorus into Host Cells. J Bacteriol. 1974;118: 663–680. doi: 10.1128/jb.118.2.663-680.1974 4208138 PMC246802

[49] Kuru E, Lambert C, Rittichier J, Till R, Ducret A, Derouaux A, et al. Fluorescent D-amino-acids reveal bi-cellular cell wall modifications important for Bdellovibrio bacteriovorus predation. Nat Microbiol. 2017;2: 1648–1657. doi: 10.1038/s41564-017-0029-y 28974693 PMC5705579

[50] Makowski Ł, Trojanowski D, Till R, Lambert C, Lowry R, Sockett RE, et al. Dynamics of chromosome replication and its relationship to predatory attack lifestyles in Bdellovibrio bacteriovorus. Appl Environ Microbiol. 2019. doi: 10.1128/AEM.00730-19 31076424 PMC6606864

[51] Gray KM, Ruby EG. Prey-derived signals regulating duration of the developmental growth phase of Bdellovibrio bacteriovorus. J Bacteriol. 1990;172: 4002–4007. doi: 10.1128/jb.172.7.4002-4007.1990 2193927 PMC213385

[52] Fenton AK, Kanna M, Woods RD, Aizawa S-I, Sockett RE. Shadowing the actions of a predator: backlit fluorescent microscopy reveals synchronous nonbinary septation of predatory Bdellovibrio inside prey and exit through discrete bdelloplast pores. J Bacteriol. 2010;192: 6329–6335. doi: 10.1128/JB.00914-10 20935099 PMC3008530

[53] Santin YG, Lamot T, Raaphorst R van, Kaljević J, Laloux G. Modulation of prey size reveals adaptability and robustness in the cell cycle of an intracellular predator. Curr Biol. 2023. doi: 10.1016/j.cub.2023.04.059 37207648

[54] Harding CJ, Huwiler SG, Somers H, Lambert C, Ray LJ, Till R, et al. A lysozyme with altered substrate specificity facilitates prey cell exit by the periplasmic predator Bdellovibrio bacteriovorus. Nat Comms. 2020;11: 4817. doi: 10.1038/s41467-020-18139-8 32968056 PMC7511926

[55] Ireton K, Gunther N 4th, Grossman AD. spo0J is required for normal chromosome segregation as well as the initiation of sporulation in Bacillus subtilis. J Bacteriol. 1994;176: 5320–5329. doi: 10.1128/jb.176.17.5320-5329.1994 8071208 PMC196717

[56] Mohl DA, Easter J, Gober JW. The chromosome partitioning protein, ParB, is required for cytokinesis in Caulobacter crescentus. Mol Microbiol. 2001;42: 741–755. doi: 10.1046/j.1365-2958.2001.02643.x 11722739

[57] Jakimowicz D, Chater K, Zakrzewska-Czerwínska J. The ParB protein of Streptomyces coelicolor A3(2) recognizes a cluster of parS sequences within the origin-proximal region of the linear chromosome. Mol Microbiol. 2002;45: 1365–77. doi: 10.1046/j.1365-2958.2002.03102.x 12207703

[58] Donovan C, Schwaiger A, Kramer R, Bramkamp M. Subcellular Localization and Characterization of the ParAB System from Corynebacterium glutamicum. J Bacteriol. 2010;192: 3441–3451. doi: 10.1128/JB.00214-10 20435732 PMC2897671

[59] Deghelt M, Mullier C, Sternon J-F, Francis N, Laloux G, Dotreppe D, et al. G1-arrested newborn cells are the predominant infectious form of the pathogen Brucella abortus. Nat Comms. 2014;5: 4366. doi: 10.1038/ncomms5366 25006695 PMC4104442

[60] Dubarry N, Willis CR, Ball G, Lesterlin C, Armitage JP. In Vivo Imaging of the Segregation of the 2 Chromosomes and the Cell Division Proteins of Rhodobacter sphaeroides Reveals an Unexpected Role for MipZ. mBio. 2019;10. doi: 10.1128/mBio.02515-18 30602584 PMC6315104

[61] Ehrle HM, Guidry JT, Iacovetto R, Salisbury AK, Sandidge DJ, Bowman GR. Polar Organizing Protein PopZ Is Required for Chromosome Segregation in Agrobacterium tumefaciens. J Bacteriol. 2017;199. doi: 10.1128/JB.00111-17 28630129 PMC5553026

[62] Pinho MG, Errington J. A divIVA null mutant of Staphylococcus aureus undergoes normal cell division. FEMS Microbiol Lett. 2004;240: 145–149. doi: 10.1016/j.femsle.2004.09.038 15522501

[63] Minnen A, Attaiech L, Thon M, Gruber S, Veening J-W. SMC is recruited to oriC by ParB and promotes chromosome segregation in Streptococcus pneumoniae. Mol Microbiol. 2011;81: 676–688. doi: 10.1111/j.1365-2958.2011.07722.x 21651626

[64] Harms A, Treuner-Lange A, Schumacher D, Søgaard-Andersen L. Tracking of Chromosome and Replisome Dynamics in Myxococcus xanthus Reveals a Novel Chromosome Arrangement. PLoS Genet. 2013;9: e1003802. doi: 10.1371/journal.pgen.1003802 24068967 PMC3778016

[65] Yamaichi Y, Fogel MA, Waldor MK. par genes and the pathology of chromosome loss in Vibrio cholerae. Proceedings of the National Academy of Sciences. 2007;104: 630–635. doi: 10.1073/pnas.0608341104 17197419 PMC1760642

[66] Milner DS, Till R, Cadby I, Lovering AL, Basford SM, Saxon EB, et al. Ras GTPase-like protein MglA, a controller of bacterial social-motility in Myxobacteria, has evolved to control bacterial predation by Bdellovibrio. PLoS Genet. 2014;10: e1004253. doi: 10.1371/journal.pgen.1004253 24721965 PMC3983030

[67] Vecchiarelli AG, Neuman KC, Mizuuchi K. A propagating ATPase gradient drives transport of surface-confined cellular cargo. Proc Natl Acad Sci USA. 2014;111: 4880–4885. doi: 10.1073/pnas.1401025111 24567408 PMC3977271

[68] Lim HC, Bernhardt TG. A PopZ-linked apical recruitment assay for studying protein–protein interactions in the bacterial cell envelope. Mol Microbiol. 2019;112: 1757–1768. doi: 10.1111/mmi.14391 31550057 PMC7218919

[69] Karunker I, Rotem O, Dori-Bachash M, Jurkevitch E, Sorek R. A Global Transcriptional Switch between the Attack and Growth Forms of Bdellovibrio bacteriovorus. Plos One. 2013;8: e61850. doi: 10.1371/journal.pone.0061850 23613952 PMC3627812

[70] Milner DS, Ray LJ, Saxon EB, Lambert C, Till R, Fenton AK, et al. DivIVA Controls Progeny Morphology and Diverse ParA Proteins Regulate Cell Division or Gliding Motility in Bdellovibrio bacteriovorus. Front Microbiol. 2020;11: 542. doi: 10.3389/fmicb.2020.00542 32373080 PMC7186360

[71] Guilhas B, Walter J-C, Rech J, David G, Walliser NO, Palmeri J, et al. ATP-driven separation of liquid phase condensates in bacteria. Mol Cell. 2020;79: 293–303.e4. doi: 10.1016/j.molcel.2020.06.034 32679076

[72] Hu L, Rech J, Bouet J-Y, Liu J. Spatial control over near-critical-point operation ensures fidelity of ParABS-mediated DNA partition. Biophys J. 2021;120: 3911–3924. doi: 10.1016/j.bpj.2021.08.022 34418367 PMC8511131

[73] Jalal AS, Tran NT, Le TB. ParB spreading on DNA requires cytidine triphosphate in vitro. Elife. 2020;9: e53515. doi: 10.7554/eLife.53515 32077854 PMC7053999

[74] Donczew M, Mackiewicz P, Wróbel A, Flärdh K, Zakrzewska-Czerwińska J, Jakimowicz D. ParA and ParB coordinate chromosome segregation with cell elongation and division during Streptomyces sporulation. Open Biology. 2016;6: 150263. doi: 10.1098/rsob.150263 27248800 PMC4852455

[75] Jakimowicz D, Żydek P, Kois A, Zakrzewska-Czerwińska J, Chater KF. Alignment of multiple chromosomes along helical ParA scaffolding in sporulating Streptomyces hyphae. Mol Microbiol. 2007;65: 625–641. doi: 10.1111/j.1365-2958.2007.05815.x 17635186

[76] Szafran MJ, Małecki T, Strzałka A, Pawlikiewicz K, Duława J, Zarek A, et al. Spatial rearrangement of the Streptomyces venezuelae linear chromosome during sporogenic development. Nat Comms. 2021;12: 1–15. doi: 10.1038/s41467-021-25461-2 34471115 PMC8410768

[77] Yamaichi Y, Niki H. Active segregation by the Bacillus subtilis partitioning system in Escherichia coli. Proc Natl Acad Sci. 2000;97: 14656–14661. doi: 10.1073/pnas.97.26.14656 11121066 PMC18974

[78] Jalal ASB, Tran NT, Wu LJ, Ramakrishnan K, Rejzek M, Gobbato G, et al. CTP regulates membrane-binding activity of the nucleoid occlusion protein Noc. Mol Cell. 2021. doi: 10.1016/j.molcel.2021.06.025 34270916 PMC8429893

[79] Omnus DJ, Fink MJ, Szwedo K, Jonas K. The Lon protease temporally restricts polar cell differentiation events during the Caulobacter cell cycle. Elife. 2021;10: e73875. doi: 10.7554/eLife.73875 34693909 PMC8545394

[80] Remy O, Lamot T, Santin Y, Kaljević J, Pierpont C de, Laloux G. An optimized workflow to measure bacterial predation in microplates. Star Protoc. 2022;3: 101104. doi: 10.1016/j.xpro.2021.101104 35098160 PMC8783149

[81] Karimova G, Ullmann A, Ladant D. A bacterial two-hybrid system that exploits a cAMP signaling cascade in Escherichia coli. Meth Enzymol. 2000;328: 59–73. doi: 10.1016/s0076-6879(00)28390-0 11075338

[82] Schindelin J, Arganda-Carreras I, Frise E, Kaynig V, Longair M, Pietzsch T, et al. Fiji: an open-source platform for biological-image analysis. Nat Methods. 2012;9: 676–682. doi: 10.1038/nmeth.2019 22743772 PMC3855844

[83] Paintdakhi A, Parry B, Campos M, Irnov I, Elf J, Surovtsev I, et al. Oufti: an integrated software package for high-accuracy, high-throughput quantitative microscopy analysis. Mol Microbiol. 2016;99: 767–777. doi: 10.1111/mmi.13264 26538279 PMC4752901

[84] Hocking J, Priyadarshini R, Takacs CN, Costa T, Dye NA, Shapiro L, et al. Osmolality-dependent relocation of penicillin-binding protein PBP2 to the division site in Caulobacter crescentus. J Bacteriol. 2012; 1–51. doi: 10.1128/jb.00260-12 22505677 PMC3370875

[85] Gray WT, Govers SK, Xiang Y, Parry BR, Campos M, Kim S, et al. Nucleoid size scaling and intracellular organization of translation across bacteria. Cell. 2019;177: 1632–1648.e20. doi: 10.1016/j.cell.2019.05.017 31150626 PMC6629263

[86] RCoreTeam. R: A language and environment for statistical computing. 2021. Available: https://www.R-project.org/

