# Supplementary figures and images for: Cell cycle-dependent organization of a bacterial centromere through multi-layered regulation of the ParABS system
Source: PLoS Genet. 2023 Sep 21;19(9):e1010951. doi: 10.1371/journal.pgen.1010951 (PMC10547168; doi:10.1371/journal.pgen.1010951)

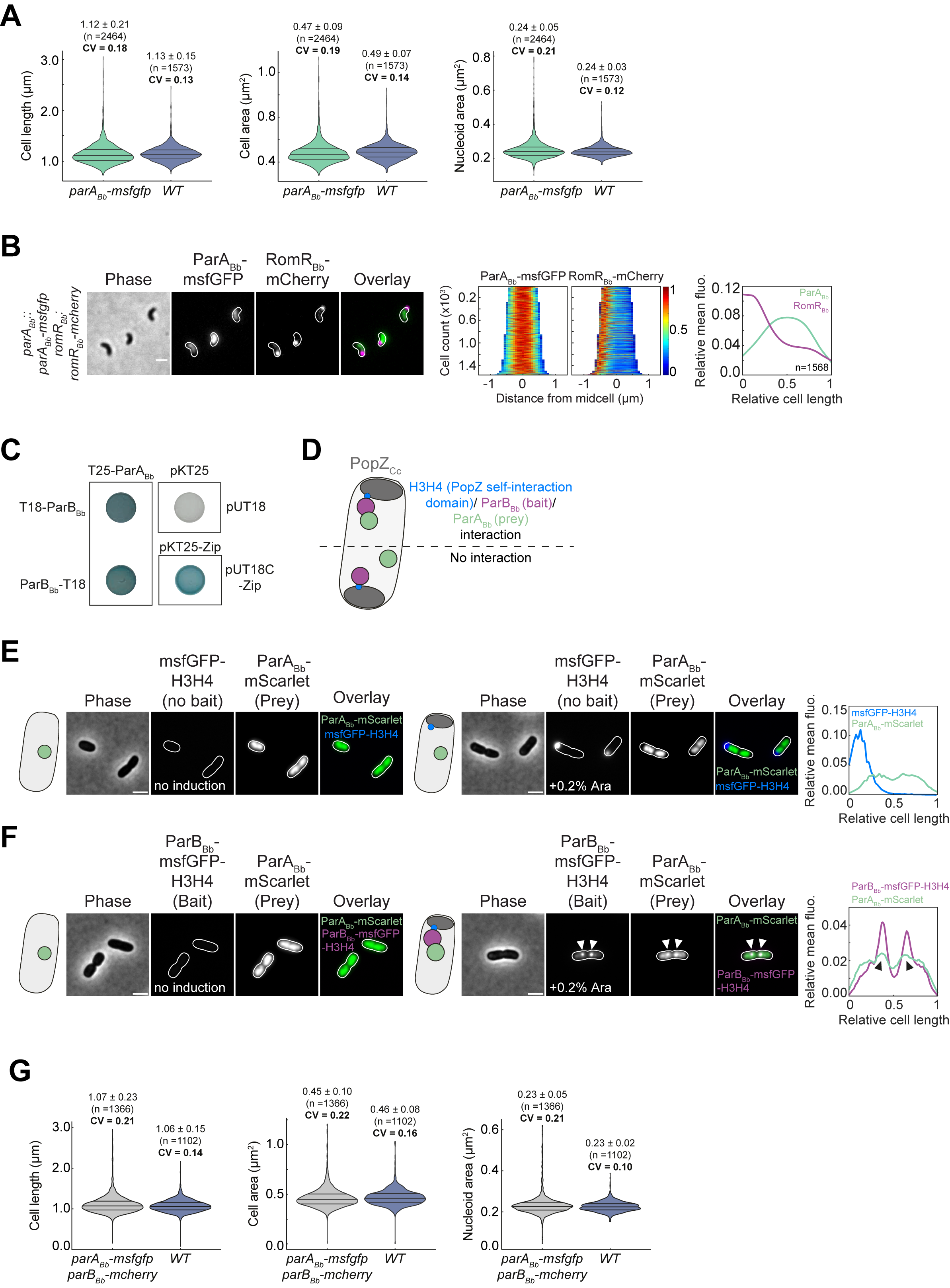

Supplement: S1 Fig — Related to Fig 1. (A) The endogenous ParABb-msfGFP fusion is functional. Violin plots of cell length, cell area, and nucleoid area distributions in parABb::parABb-msfgfp (GL2134) and WT B. bacteriovorus strains, measured from cells in Fig 1E. The lines indicate the 25, 50, and 75 percent quantiles from bottom to top. Mean, standard deviation and coefficient of variation (CV) values are shown on top of the corresponding plot. n indicate the number of cells analyzed in a representative experiment. (B) ParABb is not oriented towards any cell pole in AP cells. Left to right: representative phase contrast and fluorescence images of AP cells of parABb::parABb-msfgfp romR::romR-mcherry strain (GL2155); demographs of the corresponding fluorescent signals in the same cells sorted by length and oriented based on RomR-mCherry signal intensity; heatmaps represent relative fluorescence intensities; mean pole-to-pole profiles of relative fluorescence intensity of the corresponding fusions in the same cells. Scale bar is 1 μm. (C) ParABb and ParBBb interact in a bacterial two-hybrid assay. BTH101 reporter cells producing the indicated proteins fused to the T18 or T25 adenylate cyclase domain were spotted on X-gal agar plates supplemented with IPTG. Interaction between two proteins results in blue colony color. The Zip-Zip interaction serves as a positive control. (D) Schematic representation of the POLAR assay. POLAR takes advantage of PopZ from C. crescentus (grey), which spontaneously forms clusters at the cell poles or septa when produced in E. coli. The unlabeled PopZ is produced along with an msfGFP-H3H4 fusion to the bait protein of choice (here ParBBb), depicted in magenta; H3H4 is a PopZ self-interacting domain (shown in blue) that is sufficient to draw the bait protein to PopZ clusters. The prey protein (ParABb) is depicted in green. The prey protein is recruited to the PopZ-bait cluster if prey and bait proteins interact (upper half of the cell), whereas a lack of intera [file pgen.1010951.s001.tif]

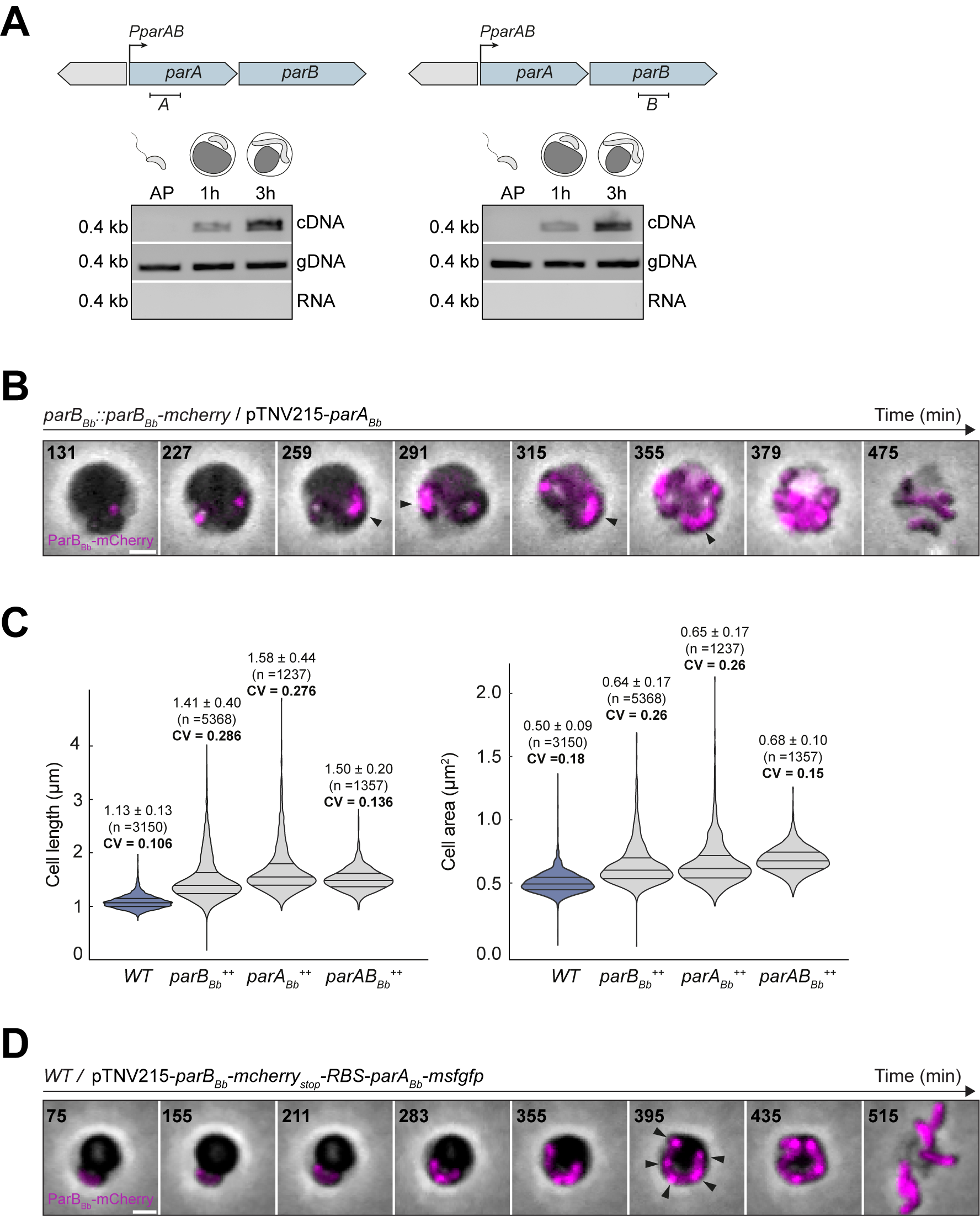

Supplement: S2 Fig — Related to Fig 2. (A) Expression of the parABb and parBBb genes, individually, follow the same biphasic pattern as the fragment covering both genes. RT-PCR experiment as in Fig 2A using primer pairs hybridizing within the parABb (left) or the parBBb gene (right). (B) Overexpression of parABb leads to chromosome segregation defects. B. bacteriovorus strain parBBb::parBBb-mcherry / pTNV215-parABb (GL2129) was mixed with prey and imaged in time-lapse after 120 min with 8-min intervals. Left: phase contrast and fluorescence images of selected time points; arrowhead points to an altered ParBBb-mCherry behavior (patches instead of well-separated foci) during the cell cycle. (C) Overexpression of parBBb or parABb leads to pronounced phenotypes, largely rescued by overexpression of both. Violin plots of cell length, cell area, and nucleoid area distributions in WT / pTNV215-parBBb (parBBb ++, GL1261); WT / pTNV215-parABb (parABb ++, GL1460); WT / pTNV215-parBBb-parABb (parABBb ++, GL1004) and WT B. bacteriovorus from cells in Fig 2B. The lines indicate the 25, 50, and 75 percent quantiles from bottom to top. Mean, standard deviation and coefficient of variation (CV) values are shown on top of the corresponding plot. n indicate the number of cells analyzed in a representative experiment. (D) Overexpression of both parABb and parBBb has no obvious effect on ori segregation. B. bacteriovorus strain WT / pTNV215-parBBb-mcherrystop-RBS-parABb-msfgfp (GL1004) was mixed with prey and imaged in time-lapse after 75 min with 8-min intervals. Left: phase contrast and fluorescence images of selected time points; arrowheads point to well-distributed ParBBb-mCherry foci (which mark chromosomal ori) during the cell cycle; ParABb-msfGFP not shown for simplicity. All experiments were performed at least twice. Scale bars are 1 μm. (TIF) [file pgen.1010951.s002.tif]

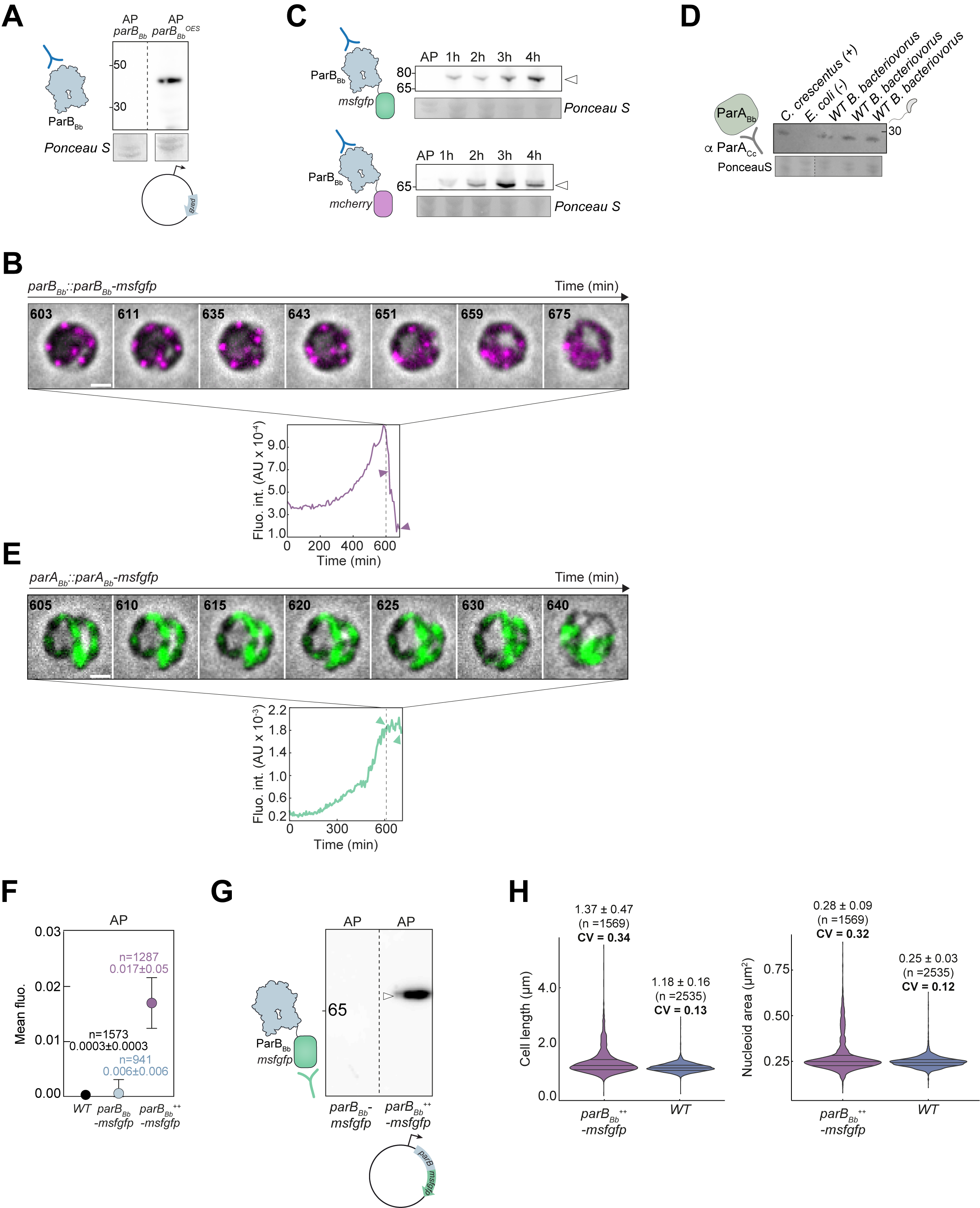

Supplement: S3 Fig — Related to Fig 3. (A) The anti-ParBBb antibody detects overproduced (right) but not native levels (right) of untagged ParBBb in AP; control experiment for Fig 3A. Western blots of whole-cell protein extracts from AP cells of WT B. bacteriovorus and a strain constitutively producing the untagged ParBBb (GL1261) were probed with an anti-ParBBb antibody represented in the schematics on the left. (B) Same as in Fig 3B for the strain natively producing ParBBb-msfGFP (GL1654). (C) The protein levels of natively produced ParBBb-msfGFP or ParBBb-mCherry detected with the same anti-ParBBb antibody compare with the endogenous untagged ParBBb profile during a synchronized B. bacteriovorus cell cycle. Western blots of whole-cell protein extracts from B. bacteriovorus strains parBBb::parBBb-msfgfp (GL1654) and parBBb::parBBb-mcherry (GL906) were probed with an anti-ParBBb antibody, as represented in the schematics. Protein samples are isolated at time points throughout the predatory cell cycle: AP, 1h, 2h, 3h, and 4h after mixing with prey. Arrowhead indicates detected ParBBb protein during the growth phase. Ponceau staining of the same membranes (where bands were most visible, ~30-50 kDa) is illustrated below each blot as a loading control. Molecular weight markers (kDa) are shown on the side. (D) ParABb protein is detected in the AP. Western blots of whole-cell protein extracts from Caulobacter crescentus (positive control), E. coli MG1655 (negative control), and WT B. bacteriovorus (in triplicates), were probed with an anti-ParACc antibody, as represented in the schematics on the left. Untagged ParABb (~29 kDa) is detected in the AP in all three samples. Ponceau staining of the same membranes (where bands were most visible, ~30-50 kDa) is illustrated below each blot as a loading control. Molecular weight markers (kDa) are shown on the side. (E) Same as in Fig 3B for the strain natively producing ParABb-msfGFP (GL2134). (F) Mean msfGFP fluorescence measured for AP cells of WT, [file pgen.1010951.s003.tif]

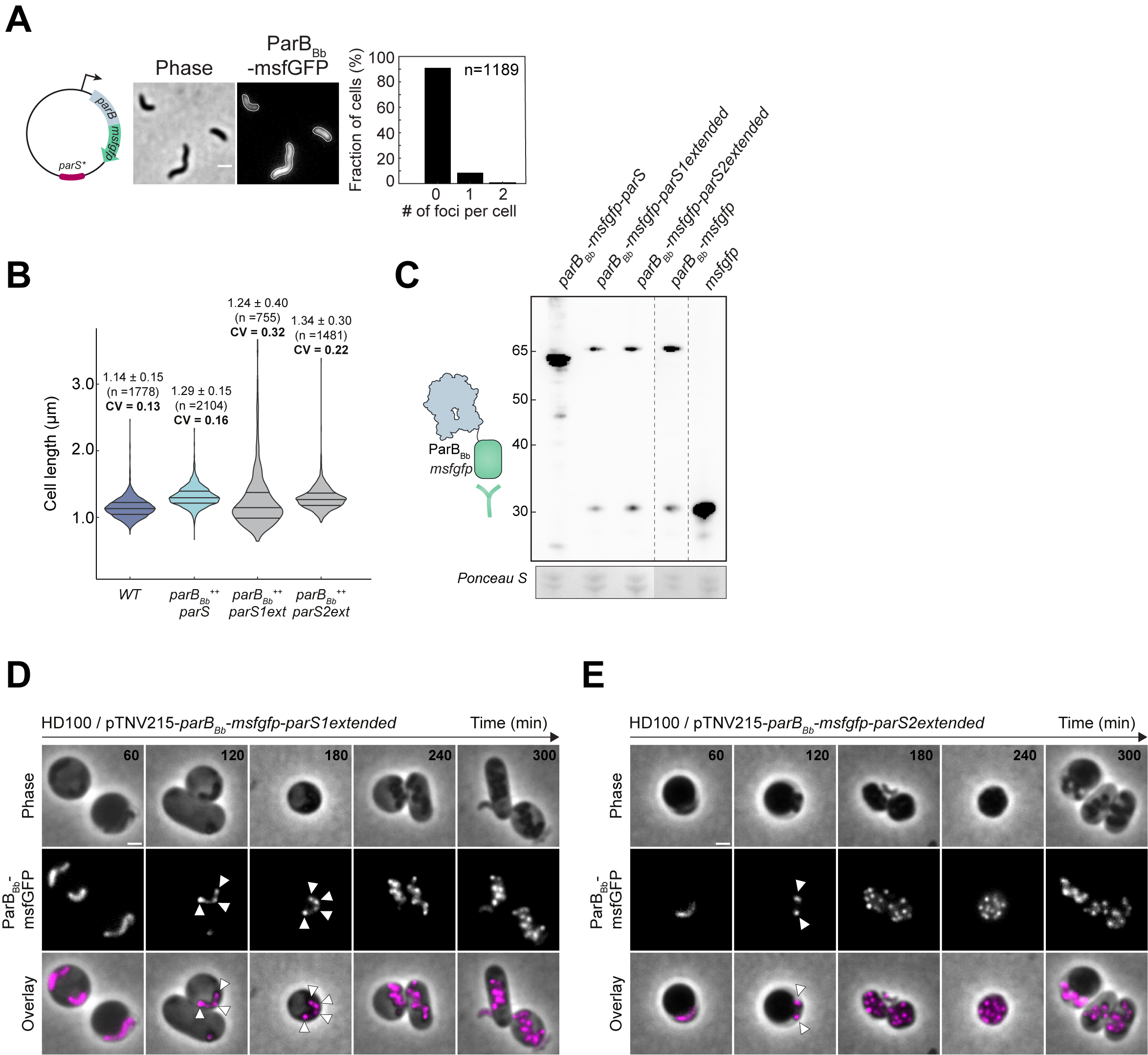

Supplement: S5 Fig — Related to Fig 5. (A) From left to right: representative phase contrast and fluorescence images of AP cells of WT B. bacteriovorus constitutively producing ParBBb-msfGFP from a plasmid carrying a mutated –parSBb (parSBb*, GL1541). Right: histogram representing the percentage of cells with zero, one, or two ParBBb-msfGFP foci in the same strain. (B) Only the cytosolic excess ParBBb is toxic for B. bacteriovorus AP cells. Violin plots of cell length for the cells in Fig 5A–5C and WT B. bacteriovorus. The lines indicate the 25, 50, and 75 percent quantiles from bottom to top. Mean, standard deviation, and coefficient of variation (CV) values are shown on top of the corresponding plot. n indicate the number of cells analyzed in a representative experiment; all experiments were performed at least twice. (C) Diffuse ParBBb-msfGFP signal in AP cells carrying plasmidic parSextended sequences is not due to protein instability. Western blots of whole-cell protein extracts from AP cells of B. bacteriovorus WT / pTNV215-parBBb-msfgfp-parS (GL1750, AP, fluorescent focus), WT / pTNV215-parBBb-msfgfp-parS1extended (GL1749, AP, diffuse signal), WT / pTNV215-parBBb-msfgfp-parS1extended (GL1925, AP, diffuse signal), WT / pTNV215-parBBb-msfgfp (GL1003, AP, diffuse signal) and WT / pTNV215-msfgfp (GL1208, control) strains were probed with an anti-msfGFP antibody, as represented in the schematics on the left. (D-E) ParBBb-msfGFP is able to form foci in the growth phase in strains carrying plasmidic parSextended sequences. Time-course experiment with strains (D) GL1749 (WT / pTNV215-parBBb-msfgfp-parS1extended) and (E) GL1825 (WT / pTNV215-parBBb-msfgfp-parS2extended). Cells were mixed with prey and imaged every hour. Phase contrast and fluorescence images of selected timepoints are shown; the white arrowheads indicate the appearance of ParBBb-msfGFP foci. (TIF) [file pgen.1010951.s005.tif]

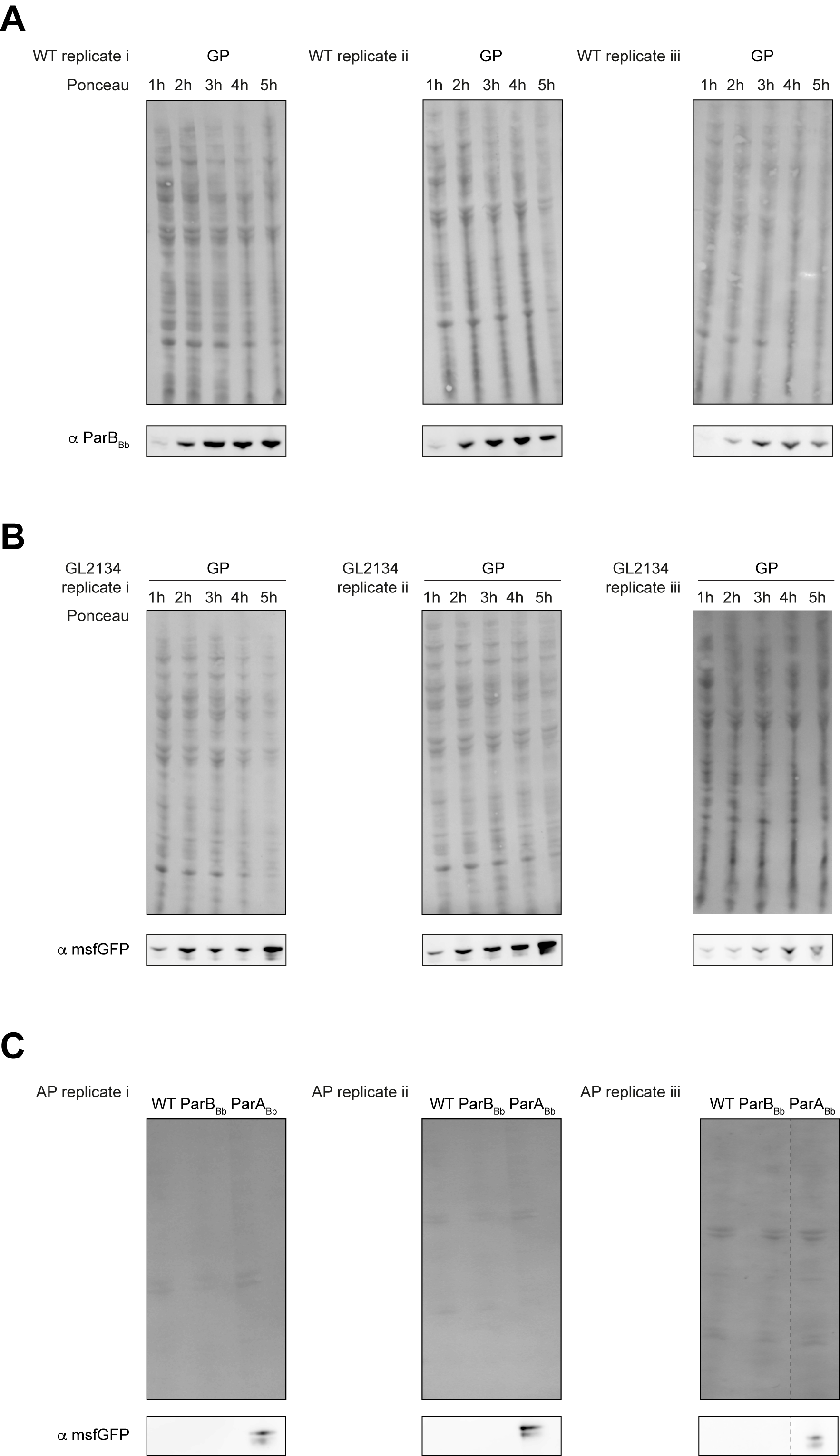

Supplement: S6 Fig — (A) Top: membranes used in Fig 3A stained with PonceauS, in triplicates. Bottom: Western Blot signal from anti-ParBBb antibody is shown in triplicates. (B) Same as in (A) for samples used in Fig 3C. (C) Same as in (A) for samples used in Fig 3D. In all cases, PonceauS staining indicates that comparable amounts of total protein were loaded in each set of samples. Unprocessed images and full lanes of PonceauS staining are used for ImageQuantTL quantification, as a proxy for total loaded proteins in order to normalize Western blot band intensities (see Methods). (TIF) [file pgen.1010951.s006.tif]
