# Supplementary material for: Cell cycle-dependent organization of a bacterial centromere through multi-layered regulation of the ParABS system
Source: PLoS Genet. 2023 Sep 21;19(9):e1010951. doi: 10.1371/journal.pgen.1010951 (PMC10547168; doi:10.1371/journal.pgen.1010951)
Supplement: S4 Fig — Related to Fig 4. (A) parSBb sequence matches with the consensus. Top: parS sequence from B. bacteriovorus. Bottom: a sequence logo generated by WebLogo 3.0 [1], using parS sequence alignments from [5] as input. (B) ParBBb is a CTPase. Continuous monitoring of the released inorganic phosphate (Pi) by recording the absorbance at 360 nm overtime at 25°C. The NTP hydrolysis of ParBBb was also monitored in the presence of ATP, GTP, or UTP, with a 22 bp parSBb DNA duplex. (C) ParBBb does not cluster without parS or on non-cognate parS. Left: representative phase contrast and fluorescence images of MG1655 E. coli strain constitutively producing ParBBb-msfGFP from a plasmid carrying no parS sequence (GL1661) or the C. crescentus parS (parSCc; GL2024). Right: histograms representing the percentage of cells with zero, one, or two ParBBb-msfGFP foci in the same strains. The scale bar is 2 μm; schematics illustrate parBBb-msfgfp expression plasmids. (D) ParB from C. crescentus (ParBCc) is more promiscuous to parS binding than ParBBb. BLI analysis of the interaction between 1 μM ParBCc and a 40 bp cognate parSCc (grey) or a non-cognate parSBb (blue). ParBCc binds both parS sequences. (E) ParBCc can bind to its cognate parSCc and a non-cognate parSBb in E. coli. Left: representative phase contrast and fluorescence images of MG1655 E. coli strain constitutively producing ParBCc-msfGFP from a plasmid carrying parSCc (left, GL2025) or parSBb (right, GL2026); fluorescent foci are observed in both cases. The scale bar is 2 μm; schematics illustrate the parBBb-msfgfp expression plasmid. (PDF) [file pgen.1010951.s004.pdf]

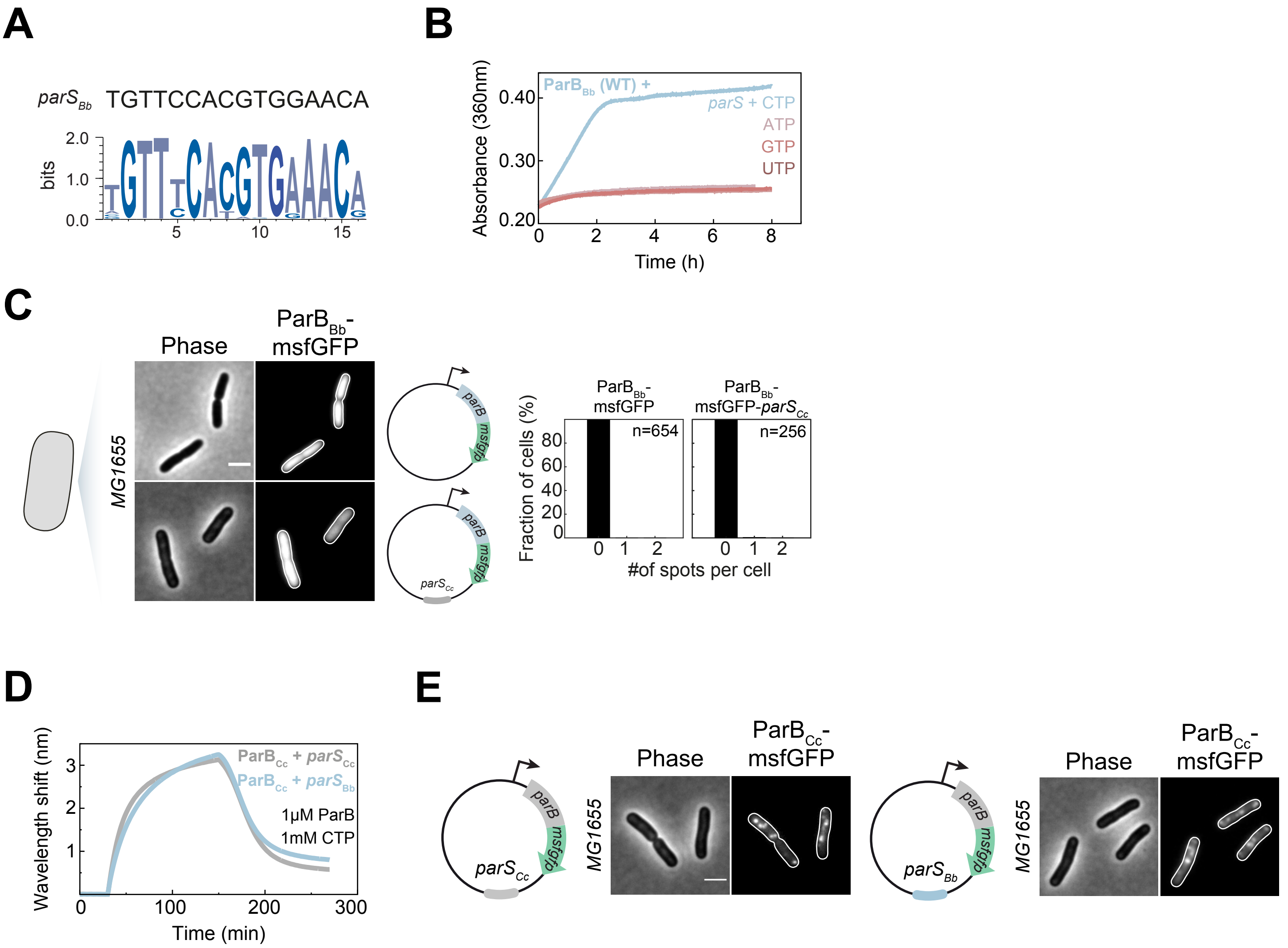

References

1. Crooks GE, Hon G, Chandonia J-M, Brenner SE. WebLogo: A Sequence Logo Generator. Genome Res. 2004;14: 1188–1190. doi:10.1101/gr.849004
2. Livny J, Yamaichi Y, Waldor MK. Distribution of Centromere-Like parS Sites in Bacteria: Insights from Comparative Genomics. J Bacteriol. 2007;189: 8693–8703. doi:10.1128/jb.01239-07
