# Supplementary material for: Cell cycle-dependent organization of a bacterial centromere through multi-layered regulation of the ParABS system
Source: PLoS Genet. 2023 Sep 21;19(9):e1010951. doi: 10.1371/journal.pgen.1010951 (PMC10547168; doi:10.1371/journal.pgen.1010951)
Supplement: S1 Table — (DOCX) [file pgen.1010951.s007.docx]

| ***Bdellovibrio bacteriovorus*** | | | |
| --- | --- | --- | --- |
| **Strains** | **Description** | **Resistance** | **Source or reference** |
| **GL734** | HD100 | / | [1] |
| **GL906** | HD100 *parB_Bb_*::*parB_Bb_-mcherry* | / | [1] |
| **GL1003** | HD100 */* pTNV215*-parB_Bb_-msfgfp* | Gm | [1] |
| **GL1004** | HD100 */* pTNV215*-parB_Bb_-mcherry_stop_-RBS-parA_Bb_-msfgfp* | Gm | [1] |
| **GL1212** | HD100 *Bd0063-0064*::*pBioFab*-*sfgfp* | / | [1] |
| **GL1261** | HD100 */* pTNV215*-parB_Bb_* | Gm | [1] |
| **GL1460** | HD100 */* pTNV215*-parA_Bb_* | Gm | This study |
| **GL1541** | HD100 */* pTNV215-*parB_Bb_-msfgfp-parS_*_* | Gm | This study |
| **GL1654** | HD100 *parB_Bb_*::*parB_Bb_-msfgfp* | / | This study |
| **GL1749** | HD100 */* pTNV215-*parB_Bb_-msfgfp-parS_1extended_* | Gm | This study |
| **GL1751** | HD100 */* pTNV215-*parB_Bb_-msfgfp-parS* | Gm | This study |
| **GL1925** | HD100 */* pTNV215-*parB_Bb_-msfgfp-parS_2extended_* | Gm | This study |
| **GL2108** | HD100 */* pTNV215*-parB_Cc_-msfgfp* | Gm | This study |
| **GL2129** | HD100 *parB_Bb_*::*parB_Bb_-mcherry /* pTNV215-*parA_Bb_* | Gm | This study |
| **GL2134** | HD100 *parA_Bb_*::*parA_Bb_-msfgfp* | / | This study |
| **GL2154** | HD100 *parB_Bb_*::*parB_Bb_-mcherry parA_Bb_::parA_Bb_-msfgfp* | / | This study |
| **GL2155** | HD100 *parA_Bb_*::*parA_Bb_-msfgfp romR::romR-mcherry* | / | This study |
| ***Caulobacter crescentus*** | | | |
| **GL01** | CB15N | Nal | Lab collection |
| ***E. coli*** | | | |
| **Strains** | **Description** | **Resistance** | **Source or reference** |
| **S17-1 λ*pir*** | Donor strain for conjugative transfer (chromosomally integrated RP4 plasmid) | Strep | Lab collection |
| **MG1655** | WT *E. coli* strain used as prey for *B. bacteriovorus* | / | Lab collection |
| **BTH101** | *E. coli* strain used for bacterial two-hybrid assay | Strep | Lab collection |
| **BL21** | *E. coli* strain used for protein purification | / | Lab collection |
| **TOP10** | *E. coli* strain used for POLAR bait plasmid cloning | / | Lab collection |
| **CC118 λpir** | *E. coli* strain used for POLAR prey plasmid cloning | / | Lab collection |
| **TB28/pAH69** | *E. coli* strain used for POLAR assay | Amp | [2] |
| **GL58** | MC4100 / pBAD18 | Amp | Lab collection |
| **GL606** | NEB5⍺ / pTNV215-*tdtomato* (*PnptII*-*tdtomato*-*RSF1010-oriT-p15A =* pMQ414 without yeast maintenance sequences) | Gm | [1] |
| **GL669** | TOP10 / pK18*mobsacB* | Kan | [1] |
| **GL831** | S17-1 λ*pir* / pK18*mobsacB*-*parB_Bb_up-parB_Bb_-msfgfp-parB_Bb_down* | Kan | [1] |
| **GL832** | S17-1 λ*pir* / pK18*mobsacB*-*parB_Bb_up-parB_Bb_-mcherry-parB_Bb_dow*n | Kan | [1] |
| **GL917** | S17-1 λpir / pTNV215-*parB_Bb_-mcherry* | Gm | [1] |
| **GL918** | S17-1 λpir / pTNV215-*parB_Bb_-msfgfp* | Gm | [1] |
| **GL1406** | S17-1 λpir / pTNV215-*parB_Bb_-msfgfp-parS* | Gm | This study |
| **GL1426** | S17-1 λpir / pTNV215-*parA_Bb_* | Gm | This study |
| **GL1513** | S17-1 λpir / pTNV215-*parB_Bb_-msfgfp-parS_mut_* | Gm | This study |
| **GL1638** | S17-1 λpir / pK18*mobsac*-*bd2761up-romR-mcherry-bd2761down* | Kan | This study |
| **GL1659** | S17-1 λpir / pTNV215-*parB_Bb_-msfgfp-par_S1extended_* | Gm | This study |
| **GL1661** | MG1655 / pTNV215-*parB_Bb_-msfgfp* | Gm | This study |
| **GL1669** | MG1655 / pTNV215-*parB_Bb_-msfgfp-parS* | Gm | This study |
| **GL1737** | MG1655 / pTNV215-*parB_Bb_-msfgfp-par_S1extended_* | Gm | This study |
| **GL1740** | BL21 / pET21a-*parB_Bb_-6xhis* | Amp | This study |
| **GL1849** | MG1655 / pTNV215-*parB_Bb_ -mcherry-parS_Cc_* | Gm | This study |
| **GL1850** | MG1655 / pTNV215-*parB_Cc_ -mcherry-parS_Cc_* | Gm | This study |
| **GL1851** | MG1655 / pTNV215-*parB_Cc_ -mcherry-parS_Bb_* | Gm | This study |
| **GL1899** | DH5⍺ / pTNV215-*parB_Bb_-msfgfp-parS_2extended_* | Gm | This study |
| **GL1900** | S17-1 λpir / pTNV215-*parB_Bb_-msfgfp-parS_2extended_* | Gm | This study |
| **GL1999** | S17-1 λpir / pK18*mobsac-bd3906up-parA_Bb_-msfgfp-bd3906down* | Kan | This study |
| **GL2024** | MG1655 / pTNV215-*parB_Bb_-msfgfp-parS_Cc_* | Gm | This study |
| **GL2025** | MG1655 / pTNV215-*parB_Cc_-msfgfp-parS_Cc_* | Gm | This study |
| **GL2026** | MG1655 / pTNV215-*parB_Cc_-msfgfp-parS_Bb_* | Gm | This study |
| **GL2067** | S17-1 λpir / pTNV215-*parB_Cc_-msfgfp* | Gm | This study |
| **GL2294** | TOP10 / pHCL150-*parB_Bb_* | Cm | This study |
| **GL2295** | CC118 λpir /pHCL147-*parA_Bb_* | Tet | This study |

S1 Table. **Strain information:** ***Bdellovibrio bacteriovorus, Caulobacter crescentus,* and *E. coli* strains used in this study.**

**Reference**

1. Kaljević J, Saaki TNV, Govers SK, Remy O, Raaphorst R van, Lamot T, et al. Chromosome choreography during the non-binary cell cycle of a predatory bacterium. Curr Biol. 2021;31: 3707-3720.e5. doi:10.1016/j.cub.2021.06.024

2. Lim HC, Bernhardt TG. A PopZ‐Linked Apical Recruitment Assay for Studying Protein‐Protein Interactions in the Bacterial Cell Envelope. Mol Microbiol. 2019. doi:10.1111/mmi.14391
