# Supplementary material for: Cell cycle-dependent organization of a bacterial centromere through multi-layered regulation of the ParABS system
Source: PLoS Genet. 2023 Sep 21;19(9):e1010951. doi: 10.1371/journal.pgen.1010951 (PMC10547168; doi:10.1371/journal.pgen.1010951)
Supplement: S2 Table — *pTNV215 is a low-copy plasmid (broad host range RSF1010 replicon). (DOCX) [file pgen.1010951.s008.docx]

| ***Bdellovibrio bacteriovorus*** | |
| --- | --- |
| **Strains** | **Construction method** |
| **GL1460** | Mating GL734 x GL1426 |
| **GL1541** | Mating GL734 x GL1513 |
| **GL1654** | Mating GL734 x GL831, allelic replacement |
| **GL1749** | Mating GL734 x GL1659 |
| **GL1751** | Mating GL734 x GL1406 |
| **GL1925** | Mating GL734 x GL1900 |
| **GL2108** | Mating GL734 x GL2067 |
| **GL2129** | Mating GL734 x GL1426 |
| **GL2134** | Mating GL734 x GL1999, allelic replacement |
| **GL2154** | Mating GL2134 x GL832, allelic replacement |
| **GL2155** | Mating GL2134 x GL1638, allelic replacement |
| **Plasmid description** | **Method of construction** |
| **pTNV215-*parB_Bb_-msfgfp-parS,* gm^R^** | Assembly of the following PCR-amplified fragments: pTNV215-*parB_Bb_-msfgfp* amplified from GL918 using primers oGL1220/oGL1221; *parS_Bb_* duplex synthesized by annealing oGL904/oGL905. |
| **pTNV215-*parA_Bb_,* gm^R^** | Assembly of the following PCR-amplified fragments: *parA_Bb_ (bd3906)* amplified from HD100 gDNA using primers oGL1304/oGL1305; pTNV215 vector amplified from pTNV215-*tdtomato* (GL606) using primers oGL451/oGL452. |
| **pTNV215-*parB_Bb_-msfgfp-parS_*_,* gm^R^** | Side-directed mutagenesis of plasmid from GL1406 using primers oGL1280/oGL1281 and following the kit instructions. |
| **pK18*mobsacB-*** ***bd2761up-romR-mcherry-bd2761down*, kan^R^** | Assembly of the following PCR-amplified fragments: *Bd2761* amplified from HD100 gDNA using primers oGL1236/oGL1104; *mcherry* amplified with oGL1146/oGL939 from GL832; *bd2761down* amplified from HD100 gDNA using primers oGL1237/oGL1238; vector pK18*mobsacB* (GL669) amplified with primers oGL331/oGL332. |
| **pTNV215-*parB_Bb_-msfgfp-parS_1extended_,* gm^R^** | Assembly of the following PCR-amplified fragments: *bd3907-parS1* amplified from HD100 using primers oGL1512/oGL1514; pTNV215-*parB_Bb_-msfgfp* vector amplified from GL918 with primers oGL1510/oGL1511. |
| **pET21a-*parB_Bb_-6xhis,* amp^R^** | Assembly of the following PCR-amplified fragments: pET21a vector amplified with oGL1586/oGL1587; *parB_Bb-_6xhis* amplified from HD100 using primers oGL1588/oGL1589. |
| **pTNV215-*parB_Bb_ -mcherry-parS_Cc,_* gm^R^** | Assembly of the following PCR-amplified fragments: pTNV215-*parB_Bb_-mcherry* vector (GL917) amplified with oGL1650/oGL1651; *parS*_Cc_ from Caulobacter C15 synthesized as a duplex in a PCR rection using primers oGL1652/oGL1653. |
| **pTNV215-*parB_Cc_ -mcherry-parS_Cc,_* gm^R^** | Assembly of the following PCR-amplified fragments: pTNV215-*parB_Bb_-mcherry-parS_Cc_* vector (GL1849) amplified with oGL489/oGL452; *parB_Cc_-mcherry* amplified from C15 genome (GL01) using primers oGL1659/oGL1660. |
| **pTNV215-*parB_Cc_ -mcherry-parS_Bb,_* gm^R^** | Assembly of the following PCR-amplified fragments: pTNV215-*parB_Bb_-mcherry-parS* vector (GL1849) amplified with oGL489/oGL452; *parB_Cc_-mcherry* amplified from C15 genome (GL01) using primers oGL1659/oGL1660. |
| **pTNV215-*parB_Bb_-msfgfp-parS_2extended,_* gm^R^** | Assembly of the following PCR-amplified fragments: pTNV215-*parB_Bb_-msfgfp* vector (GL918) amplified with oGL1510/oGL1511; *parS_2extended_* region amplified from HD100 using primers oGL1707/oGL1708. |
| **pK18*mobsacB-bd3906up-parA_Bb_-msfgfp-bd3906down,* kan^R^** | Assembly of the following PCR-amplified fragments: *Bd3906up* amplified from HD100 using primers oGL1785/oGL510; *msfgfp* amplified from GL918 using primers oGL1862/oGL299; *Bd3906down* amplified from HD100 using primers oGL638/oGL794; vector pK18*mobsacB* (GL669) amplified with primers oGL331/oGL332. |
| **pTNV215-*parB_Bb_-msfgfp-parS_Cc,_* gm^R^** | Assembly of the following PCR-amplified fragments: pTNV215-*parB_Bb_-msfgfp* vector (GL918) amplified with oGL1650/oGL1651; *parS_Cc_* from Caulobacter synthesized as a duplex in a PCR rection using primers oGL1652/oGL1653. |
| **pTNV215-*parB_Cc_-msfgfp-parS_Cc_,* gm^R^** | Assembly of the following PCR-amplified fragments: pTNV215-*parB_Cc_-mcherry-parS_Cc_* vector (GL1850) amplified with oGL1887/oGL451; *msfgfp* amplified from GL918 using primers oGL1316/oGL892. |
| **pTNV215-*parB_Cc_-msfgfp-parS_Bb_*, gm^R^** | Assembly of the following PCR-amplified fragments: pTNV215-*parB_Cc_-mcherry-parS_Bb_* vector (GL1851) amplified with oGL1887/oGL451; *msfgfp* amplified from GL918 using primers oGL1316/oGL892. |
| **pTNV215-*parB_Cc_-msfgfp,* gm^R^** | Assembly of the following PCR-amplified fragments: pTNV215-*parB_Bb_-msfgfp* vector (GL918) amplified with oGL452/oGL1146; *parB_Cc_* amplified from C15 (GL01) using primers oGL1659/oGL1660. |
| **pHCL150-*parB_Bb,_* cm^R^** | Assembly of the following PCR-amplified fragments: pHCL150 vector (kind gift from [4]) amplified with oGL2464/2465 and *parB_Bb_* amplified from HD100 using primers oGL2466/2467. |
| **pHCL147-*parA_Bb_*_,_ tet^R^** | Assembly of the following PCR-amplified fragments: pHCL147 vector (kind gift from [4]) amplified with oGL1696/1720 and *parA_Bb_* amplified from HD100 using primers oGL2468/2469. |

S2 Table. **Construction of *Bdellovibrio bacteriovorus* strains and plasmids used in this study.** *pTNV215 is a low-copy plasmid (broad host range RSF1010 replicon).
