# Supplementary material for: Cell cycle-dependent organization of a bacterial centromere through multi-layered regulation of the ParABS system
Source: PLoS Genet. 2023 Sep 21;19(9):e1010951. doi: 10.1371/journal.pgen.1010951 (PMC10547168; doi:10.1371/journal.pgen.1010951)
Supplement: S3 Table — Overlapping sequences used for cloning by DNA assembly and site-directed mutations are highlighted in black. (DOCX) [file pgen.1010951.s009.docx]

| **Primer name** | **Primer sequence (5’>3’)** |
| --- | --- |
| **oGL299** | GGCTCAGGAAGCGGCTCAGGATCCAAAGGA |
| **oGL321** | AATTCGAGCTCGGTACCCG |
| **oGL322** | GCTAGCCCAAAAAAACGGGTATG |
| **oGL331** | GGCACTGGCCGTCGTTTTACAA |
| **oGL332** | GTAATCATGTCATAGCTGTTTCCTGTGTGAAA |
| **oGL451** | GGATCCTGATACAGATTAAATCAGAACGCAG |
| **oGL452** | GAATTCTCCTCATCCTGTCTCTTGATCAG |
| **oGL489** | **ggaagcggctcaggatcc**GTGAGCAAGGGCGAGGAG |
| **oGL510** | **cctgagccgcttcctgagcc**TGCCATTTGTTCTGTCT |
| **oGL638** | GAGTACGACAGCAAATCCAT |
| **oGL794** | GTAAAACGACGGCCAGTGCCCGGATTCAGATCTTCACG |
| **oGL892** | **tctgatttaatctgtatcaggatcta**TTTGTAGAGCTCATCCATGC |
| **oGL904** | GCCGATGTTCCACGTGGAACAAGTGT |
| **oGL905** | ACACTTGTTCCACGTGGAACATCGGC |
| **oGL939** | CTTGTACAGCTCGTCCATGCC |
| **oGL1104** | **ggatcctgagccgcttcctgagcc**AATGGACTTTTCAGTTTCGCG |
| **oGL1146** | GGCTCAGGAAGCGGCTCAGG |
| **oGL1220** | GCCGATGTTCCACGTGGAACAAACCGCTTCTGCGTTCT |
| **oGL1221** | ACACTTGTTCCACGTGGAACATCATAAAACAGAATTTGCCTGGC |
| **oGL1236** | **caggaaacagctatgacatgattac**AATCCCAAAGCGGAAGACGAA |
| **oGL1237** | **gcatggacgagctgtacaagtaa**ATCCTCCGCGAAACTGAAAAGTC |
| **oGL1238** | ATGCCTGCAGGTCGACTCTACTCAGATTCATGCGGTGATTGACG |
| **oGL1280** | GCGGTTTG**cc**CCACGTGGAACATCATAAAA |
| **oGL1304** | TCTGATTTAATCTGTATCAGGATCCCTTATGCCATTTGTTCTGTC |
| **oGL1305** | TCAAGAGACAGGATGAGGAGAATTCAAATGGCAAAAACAATCTGC |
| **oGL1316** | GGCTCAGGAAGCGGCTC |
| **oGL1510** | ACAGATTAAATCAGAACGC |
| **oGL1511** | **atcaggatcta**TTTGTAGAG |
| **oGL1512** | **atgagctctacaaa**TAGATCCTGATCAGATTGTTTTTGCCAT |
| **oGL1514** | GCTTCTGCGTTCTGATTTAATCTGCGGTGAATATAGGATCC |
| **oGL1586** | TGAGATCCGGCTGCTA |
| **oGL1587** | ATGTATATCTCCTTCTTAAAGTTAAAC |
| **oGL1588** | ATAATTTTGTTTAACTTTAAGAAGGAGATATACATATGTCTGATATTGCTGTAGAATC |
| **oGL1589** | **gctttgttagcagccggatctcagtggtggtggtggtggtg**GGATCCTGAGCCGCTTCCTGAGCC**ctgccatccttctttaagc** |
| **oGL1650** | **acact**TGTTTCACGTGAAACA**tcataaaacagaatttgcctggc** |
| **oGL1651** | **gccga**TGTTTCACGTGAAACA**agaccgcttctgcgttct** |
| **oGL1652** | **acact**TGTTTCACGTGAAACA**tcggc** |
| **oGL1653** | **gccga**TGTTTCACGTGAAACA**agtgt** |
| **oGL1659** | **tcaagagacaggatgaggagaattc**ATGGAGTCCGTCGTGGT |
| **oGL1660** | **tgctcac**GGATCCTGAGCCGCTTCCGATCCCGCGCGTCAGTCGGTT |
| **oGL1696** | TCTGGTCTCGAGGGTCCG |
| **oGL1707** | **atgagctctacaaa**TAGATCCTGATGCAAAGAAAAGCTGGGA |
| **oGL1708** | **gcttctgcgttctgatttaatct**GGGCTGGCGAATATTTCCA |
| **oGL1720** | CATATGTATATCTCCTTCTTAAAGTTAAAC |
| **oGL1785** | **cacacaggaaacagctatgacatgatta**CGAGGTACTGCTTTTCCT |
| **oGL1862** | **cgccgatggatttgctgtcgtactccta**TTTGTAGAGCTCATCCA |
| **oGL1887** | TGGATCCTGAGCCGCTTCCTGAGCCGATCCCGCGCGTCAGT |
| **oGL2464** | CTCGAGGGTGGAGGCTC |
| **oGL2465** | CATATGTATATCTCCTTCTTAAATCTAGACAGCG |
| **oGL2466** | **agatttaagaaggagatatacatatg**TCTGATATTGCTGTAGAATCCTCAAACAAG |
| **oGL2467** | **gcctccaccctcgag**CTGCCATCCTTCTTTAAGCCTATC |
| **oGL2468** | **ctttaagaaggagatatacatatg**GCAAAAACAATCTGCATAGCGAATC |
| **oGL2468** | **accctcgagaccaga**TGCCATTTGTTCTGTCTGTGG |
| **oGL2579** | AAGAGTTCTTTTGATCGACATGGACC |
| **oGL2580** | CGGTATTCAACAGCTGACTAAGCC |
| **oGL2581** | AGAACATTTGAGAAAGAGCCGCTT |
| **oGL2582** | TTTCCAGAGATCATCTCTTTCACAGAGT |
| **180 bp_*parS_Bb_*** | cgccagggttttcccagtcacgacgttgtaaaacgacggccagaattcgcaattcgattattgttga**TGTTCCACGTGGAACA**tcacgcgatgataacgactctatcattgatagagtgttctctccacgggatccccaggcatgcaagcttggcgtaatcatggtcatagctgtttcct **(*parS_Bb_* in bold)** |
| **40 bp *parS_Bb_*** | ttcgattattgttga**TGTTCCACGTGGAACA**tcacgcgat **(*parS_Bb_* in bold)** |
| **170 bp_*parS_Cc_,***  [1] | cgccagggttttcccagtcacgacgttgtaaaacgacggccagtgaattcgagctcggtacccgcaggaggacgtagggtaggggga**TGTTTCACGTGAAACA**ggggatcctctagagtcgacctgcaggcatgcaagcttggcgtaatcatggtcatagctgtttcct **(*parS_Cc_* site in bold)** |

Table S3. **Oligos used in this study.** **Overlapping sequences used for cloning by DNA assembly and site-directed mutations are highlighted in black.**

**Reference**

1. Jalal AS, Tran NT, Stevenson CE, Chimthanawala A, Badrinarayanan A, Lawson DM, et al. A CTP-dependent gating mechanism enables ParB spreading on DNA. Elife. 2021;10: e69676. doi:10.7554/elife.69676
